# The metabolic function of pyruvate kinase M2 regulates reactive oxygen species production and microbial killing by neutrophils

**DOI:** 10.1038/s41467-023-40021-6

**Published:** 2023-07-17

**Authors:** Juliana Escher Toller-Kawahisa, Carlos Hiroji Hiroki, Camila Meirelles de Souza Silva, Daniele Carvalho Nascimento, Gabriel Azevedo Públio, Timna Varela Martins, Luis Eduardo Alves Damasceno, Flávio Protásio Veras, Paula Ramos Viacava, Fábio Yuji Sukesada, Emily Anne Day, Alessia Zotta, Tristram Alexander Jasper Ryan, Rodrigo Moreira da Silva, Thiago Mattar Cunha, Norberto Peporine Lopes, Fernando de Queiroz Cunha, Luke Anthony John O’Neill, José Carlos Alves-Filho

**Affiliations:** 1 Department of Pharmacology, Ribeirao Preto Medical School, University of Sao Paulo, Ribeirao Preto, Brazil; 2 Center for Research in Inflammatory Diseases, Ribeirao Preto Medical School, University of Sao Paulo, Ribeirao Preto, Brazil; 3 School of Biochemistry and Immunology, Trinity Biomedical Science Institute, Trinity College Dublin, Dublin, Ireland; 4 NPPNS, Department of Biomolecular Sciences, School of Pharmaceutical Sciences of Ribeirao Preto, University of São Paulo, Ribeirao Preto, Brazil

**Keywords:** Neutrophils, Antimicrobial responses

## Abstract

Neutrophils rely predominantly on glycolytic metabolism for their biological functions, including reactive oxygen species (ROS) production. Although pyruvate kinase M2 (PKM2) is a glycolytic enzyme known to be involved in metabolic reprogramming and gene transcription in many immune cell types, its role in neutrophils remains poorly understood. Here, we report that PKM2 regulates ROS production and microbial killing by neutrophils. Zymosan-activated neutrophils showed increased cytoplasmic expression of PKM2. Pharmacological inhibition or genetic deficiency of PKM2 in neutrophils reduced ROS production and *Staphylococcus aureus* killing in vitro. In addition, this also resulted in phosphoenolpyruvate (PEP) accumulation and decreased dihydroxyacetone phosphate (DHAP) production, which is required for de novo synthesis of diacylglycerol (DAG) from glycolysis. In vivo, PKM2 deficiency in myeloid cells impaired the control of infection with *Staphylococcus aureus*. Our results fill the gap in the current knowledge of the importance of lower glycolysis for ROS production in neutrophils, highlighting the role of PKM2 in regulating the DHAP and DAG synthesis to promote ROS production in neutrophils.

## Abstract (web summary)

Neutrophil activation has been shown to rely on the pentose phosphate pathway (PPP) for NADPH generation and reactive oxygen species production. In this study, the authors identify a mechanism of neutrophil activation that is independent of the PPP but relies on the glycolytic enzyme pyruvate kinase M2 instead.

## Introduction

Neutrophils are the most abundant leukocytes in human blood and are the first immune cell type recruited into the site of infection^1^. Once activated, neutrophils produce a large amount of superoxide, the precursor of hydrogen peroxide and other reactive oxygen species (ROS), through the activation of the nicotinamide adenine dinucleotide phosphate (NADPH) oxidase complex. Defects in ROS production impair pathogen elimination by neutrophils leading to recurring infections, as seen in patients with chronic granulomatous disease^2^. The NADPH oxidase is an enzymatic complex composed of two membrane-bound (gp91-*phox*, p22-*phox*) and three cytosolic (p47-*phox*, p40-*phox*, p67-*phox*) subunits. The activation of NADPH oxidase requires the phosphorylation of these subunits, mainly performed by protein kinase C (PKC), which is activated by diacylglycerol (DAG) in a calcium-dependent manner. DAG is produced from the hydrolysis of phosphatidylinositol 4,5-bisphosphate (PIP_2_) by phospholipase C (PLC), which is activated by a wide variety of pro-inflammatory stimuli that signal mainly through G protein-coupled receptors (GPCRs)^3^. In addition, neutrophil metabolism reprogramming is essential for ROS production^4–6^.

Neutrophil metabolism relies mainly on glycolysis rather than oxidative phosphorylation (OXPHOS) for energy production since they harbour fewer mitochondria than other immune cells^7–9^. Interestingly, the inhibition of glycolysis using 2-deoxyglucose (2-DG) severely impairs phagocytosis, ROS production, and bacterial killing by neutrophils^4,5^. In this context, it is well-known that glycolysis fuels the pentose phosphate pathway (PPP) that utilises the glycolytic intermediate glucose-6-phosphate (G6P) to generate NADPH, essential for NADPH oxidase-dependent ROS production^6^. The importance of the PPP for neutrophil activation is observed in patients with glucose-6-phosphate dehydrogenase (G6PD) syndrome with recurrent infections associated with less ROS production by neutrophils^10^. Furthermore, Rossi and colleagues^11^ showed that de novo synthesis of DAG from glucose represents a substantial source of DAG in activated neutrophils. In this pathway, fructose-1,6-bisphosphate (F1,6BP) is converted into glyceraldehyde-3-phosphate (GAP) and dihydroxyacetone phosphate (DHAP) by fructose bisphosphate aldolase and the balance between GAP and DHAP is regulated by triosephosphate isomerase (TPI). DHAP is further converted to glycerol-3-phosphate (G3P) by glycerol-3-phosphate dehydrogenase. G3P is metabolised to phosphatidic acid and, subsequently, to DAG by phosphatidate phosphohydrolase^12^. However, whether DAG produced from glycolysis is important for ROS production is unknown.

The importance of the last steps of glycolysis for ROS production is less understood. The pyruvate kinase (PK) is a rate-limiting glycolytic enzyme that catalyses the last step of glycolysis, converting phosphoenolpyruvate (PEP) to pyruvate^13^. There are four PK isoforms: PKL, PKR, PKM1, and PKM2. PKL and PKR are expressed in the liver and erythrocytes, respectively, whereas PKM1 and PKM2 are expressed in many cell types, including neutrophils^14^. Particularly, PKM2 can be activated allosterically by endogenous regulators such as F1,6BP, some amino acids, and small synthetic molecules such as TEPP-46 that affect its PEP-binding affinities. In the absence of these activators, PKM2 assumes a dimeric or monomeric form, resulting in the accumulation of glycolytic intermediates. While dimeric PKM2 can translocate to the nucleus to function as a coactivator of transcriptional factors, regulating genes important for cell proliferation and glycolysis, allosterically activated tetrameric PKM2 has a high glycolytic capacity^15^. Although several reports have recently shown the importance of PKM2 in regulating the metabolic reprogramming and the immune function of macrophages and lymphocytes^16–18^, the role of PKM2 in the cellular metabolism and transcriptional activity in neutrophils remains poorly characterised.

Here, we show that ROS production in neutrophils is dependent on glycolytic flux. In this context, PKM2 regulates the DHAP and DAG levels, which activates PKC and, therefore, the NADPH oxidase complex to promote ROS production and killing activity in neutrophils. Our results suggest a role for PKM2 in neutrophil activation and highlight the importance of PKM2 in host defence and as a potential therapeutic target in neutrophil-mediated diseases.

## Results

### PKM2 expression increases in the cytoplasm of activated neutrophils

**Fig. 1:**
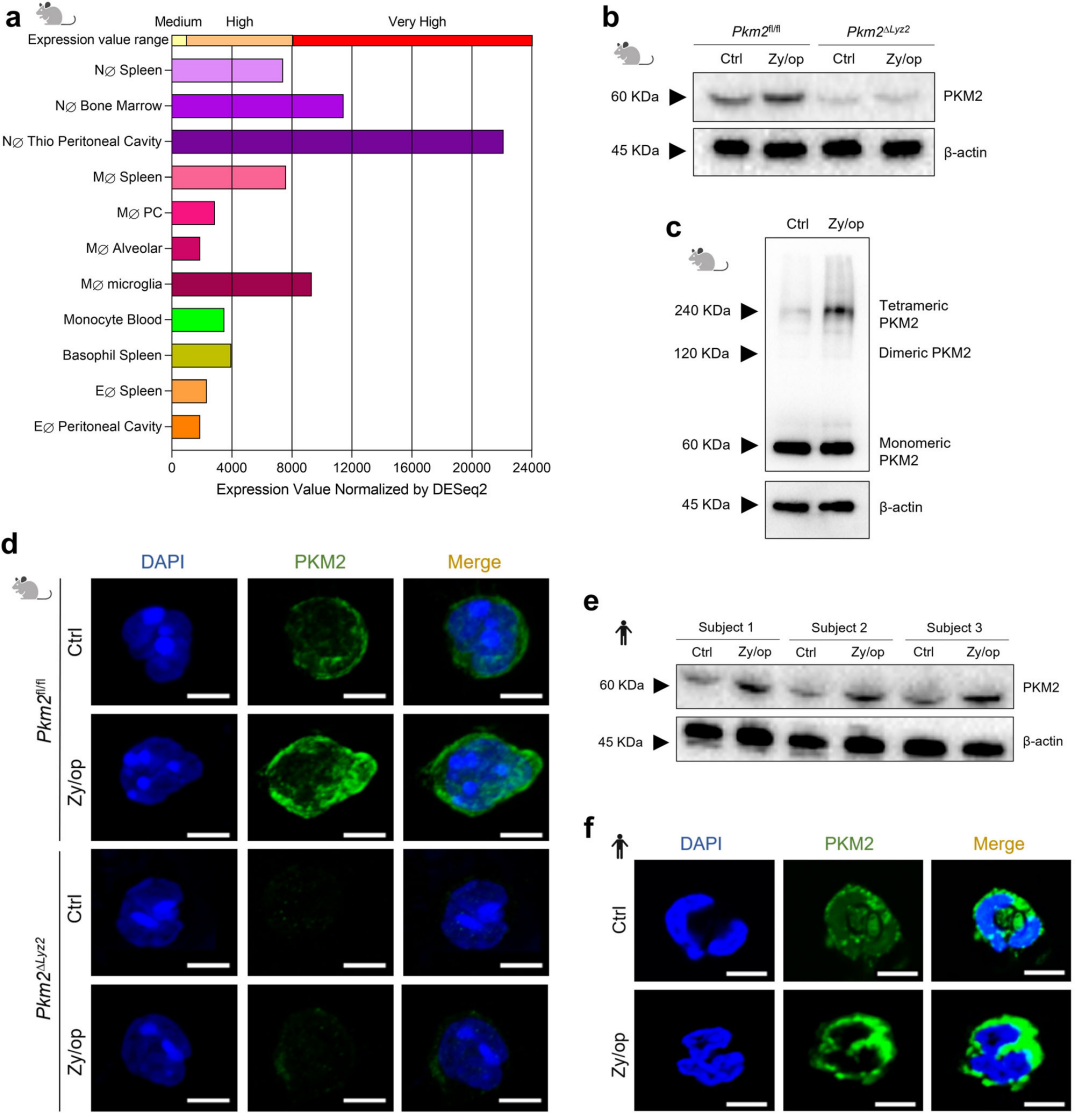
PKM2 expression increases in activated neutrophils. **a** *Pkm2* expression analysis in myeloid cells from the Immunological Genome Project (ImmGen) bulk-population RNA-seq database^14^. **b** Immunoblot analysis of PKM2 expression in BM-isolated neutrophils from wild-type (*Pkm2*^fl/fl^) and PKM2-deficient (*Pkm2*^∆*Lyz2*^) mice. Cells were activated with Zy/op (100 µg/mL) or medium only (Ctrl) for 6 h. β-actin was used as the loading control. Representative of three independent experiments. **c** Immunoblot analysis of PKM2 conformational states in murine neutrophils after activation with Zy/op for 6 h using DSS cross-linking assay. Representative of two independent experiments. **d** Confocal immunofluorescence analysis of Zy/op-activated murine neutrophils stained for PKM2 (green) and DAPI (blue). The scale bar indicates 5 μm. Representative of three independent experiments. **e** Immunoblot analysis of PKM2 expression in blood-isolated neutrophils from three healthy volunteers. Cells were activated with Zy/op for 6 h. β-actin was used as the loading control. Representative of three independent experiments. **f** Confocal immunofluorescence analysis of Zy/op-activated human neutrophils stained for PKM2 (green) and DAPI (blue). The scale bar indicates 5 μm. Representative of three independent experiments. Clip art provided by Biorender. Source data are provided in the Source data file.

We initially compared the expression of *Pkm2* between murine neutrophils and other myeloid cells by analysing data from the Immunological Genome Project^14^. We found that *Pkm2* expression is higher in neutrophils (NØ) compared to macrophages (MØ), basophils (BØ), or eosinophils (EØ) (Fig. 1a). In addition, we found that thioglycolate-elicited peritoneal neutrophils expressed higher amounts of *Pkm2* than bone marrow (BM) neutrophils, indicating that *Pkm2* expression increases in activated neutrophils (Fig. 1a). To investigate the PKM2 protein expression pattern, wild-type and PKM2-deficient neutrophils were isolated from the bone marrow of *Pkm2*^fl/fl^ and *Pkm2*^∆*Lyz2*^ mice, respectively (Supplementary Fig. 1), then activated with serum-opsonized zymosan (Zy/op) for 6 h. We found that PKM2 is constitutively expressed in control (Ctrl) neutrophils and increased when neutrophils were activated with Zy/op (Fig. 1b). Of note, PKM2-deficient neutrophils showed very low levels of PKM2 protein expression (Fig. 1b).

PKM2 exists in different conformations as a monomer, dimer, and tetramer. The stabilised tetrameric form has high glycolytic activity, converting PEP to pyruvate, and is located in the cytoplasm. The dimeric form has low glycolytic activity but can translocate into the nucleus, acting as a nuclear transcriptional coactivator regulating gene expression^15^. We found an increase in the PKM2-tetrameric form but not in the PKM2-dimeric form in Zy/op-activated neutrophils (Fig. 1c). Accordingly, using confocal microscopy, we found that PKM2 is primarily located in the cytoplasm of neutrophils (Fig. 1d). We also evaluated PKM2 expression in human neutrophils. Neutrophils were isolated from the blood of three healthy subjects and activated with Zy/op. Consistent with the data observed in murine neutrophils, human neutrophils also showed a constitutive basal expression of PKM2, increasing when neutrophils were activated with Zy/op for 6 h (Fig. 1e). Moreover, the confocal analysis showed that PKM2 was also mainly expressed in the cytoplasm of human neutrophils (Fig. 1f). Together these results show that PKM2 expression is upregulated in Zy/op-activated neutrophils and is localised mainly in the cytoplasm.

### PKM2 is required for ROS production and killing capacity by neutrophils

**Fig. 2:**
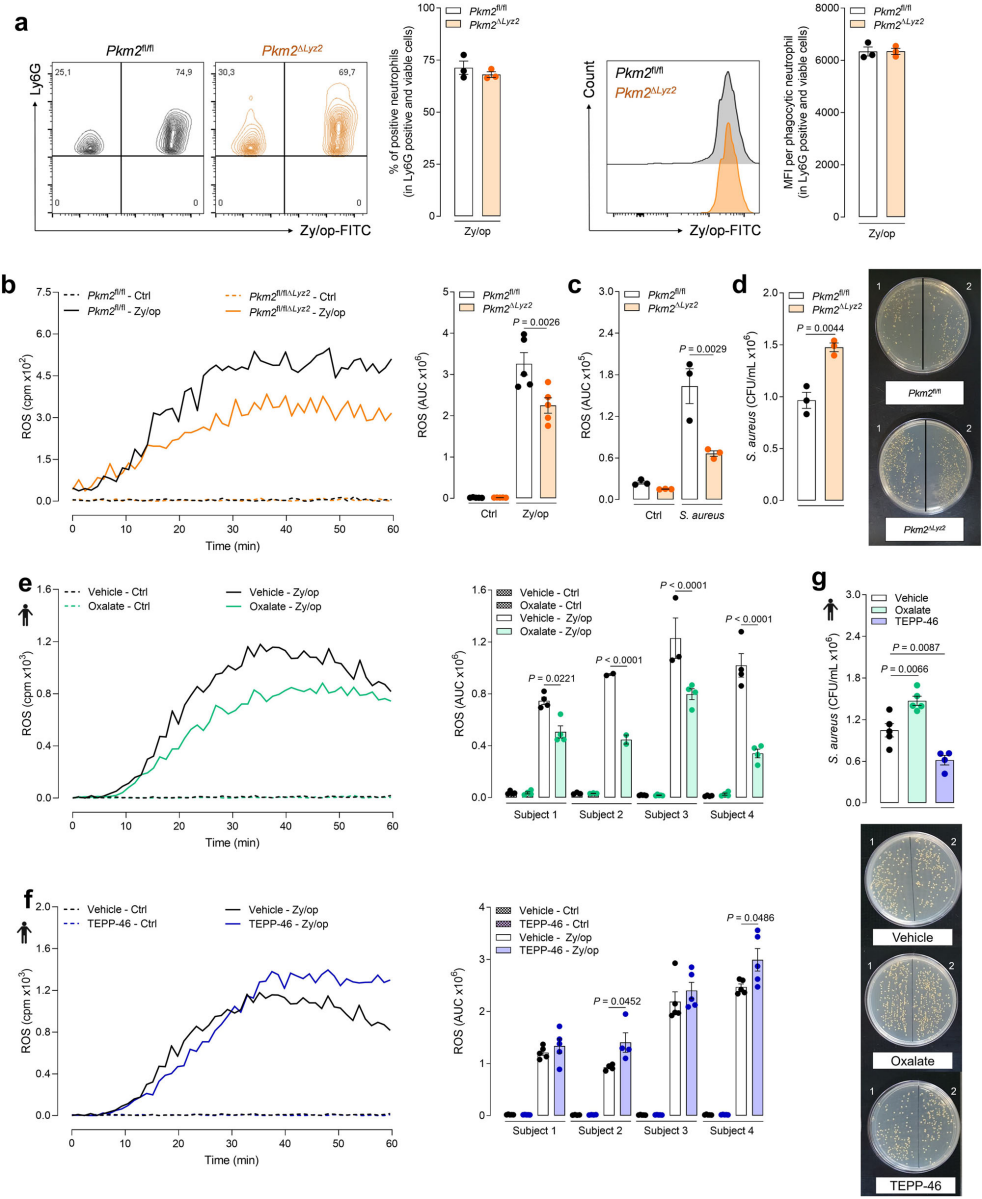
PKM2 deficiency impairs ROS production and microbial killing by neutrophils. **a** Phagocytosis of Zy/op-FITC (100 µg/mL) by wild-type (*Pkm2*^fl/fl^) and PKM2-deficient (*Pkm2*^∆*Lyz2*^) neutrophils. Representative dot plots, histograms and bar graphs showing the frequency and the median fluorescence intensity (MFI) of phagocytic cells. Gating strategies are shown in Supplementary Fig. 5a. *n* = 3 mice per group, representative of two independent experiments. **b** Kinetic measurement of ROS production by wild-type and PKM2-deficient neutrophils activated with Zy/op. Representative time-response graph and area under the curve (AUC) graph bar. *n* = 5 mice per group, representative of five independent experiments. **c** ROS production by wild-type and PKM2-deficient neutrophils exposed to *S. aureus* (2 ×10^6^) opsonized with serum. *n* = 3 mice per group, representative of two independent experiments. **d** Quantification of viable *S. aureus* recovered from lysates of wild-type and PKM2-deficient neutrophils after 2 h. *n* = 3 mice per group, representative of three independent experiments. **e** ROS production by human neutrophils pre-treated with oxalate (3 mM) for 1 h and then activated with Zy/op. Representative time-response graph and AUC graph bar. *n* = 4 donors in four independent experiments. **f** ROS production by human neutrophils pre-treated with TEPP-46 (30 µM) for 1 h and then activated with Zy/op. Representative time-response graph and AUC graph bar. *n* = 4 donors in four independent experiments. **g** Number of viable *S. aureus* recovered after 2 h from lysates of human neutrophils pre-treated with oxalate or TEPP-46 for 1 h. *n* = 5 donors in three independent experiments. Error bars are mean ± SEM. *p* values were determined by two-tailed unpaired Student’s *t*-test (**a**, **d**) or one-way ANOVA followed by Tukey’s post hoc test (**b**, **c**, **e**–**g**). Clip art provided by Biorender. Source data are provided in the Source data file.

We then conducted a series of experiments to evaluate the role of PKM2 in neutrophils’ functions. We first performed flow cytometry analysis to evaluate phagocytosis in wild-type and PKM2-deficient neutrophils activated with Zy/op-FITC. We found that PKM2 did not affect the phagocytic activity of neutrophils since the percentage of neutrophils bearing Zy/op-FITC and the amount of phagocytosed Zy/op-FITC was similar between wild-type and PKM2-deficient neutrophils (Fig. 2a). Also, there was no difference in the production of neutrophil extracellular traps (NETs) by PKM2-deficient neutrophils (Supplementary fig. 2a). We next evaluated real-time ROS production. PKM2-deficient neutrophils produced a lower amount of total ROS and superoxide than wild-type neutrophils when activated with Zy/op (Fig. 2b and Supplementary Fig. 2b). Consistently, ROS production was also impaired when PKM2-deficient neutrophils were exposed to *Staphylococcus aureus* (*S. aureus*) (Fig. 2c).

ROS is an important tool for the microbicidal activity of neutrophils^19^. Thus, we next determined the killing activity of neutrophils exposed to *S. aureus* and found that it was impaired in PKM2-deficient neutrophils (Fig. 2d). To extend the evaluation of the role of PKM2 to human neutrophils, we isolated cells from the blood of four healthy subjects and pre-treated them with oxalate, a non-selective pyruvate kinase activity inhibitor^20^, for 1 h before the activation with Zy/op. We used oxalate because there is no selective inhibitor of PKM2 enzymatic activity. We also pre-treated human neutrophils with TEPP-46, which activates PKM2 in the tetrameric form, maintaining PKM2 in the glycolytic pathway and impairing its migration into the nucleus^21^. The inhibition of PK with oxalate decreased ROS production (Fig. 2e). In contrast, activation of PKM2 with TEPP-46 increased ROS production, although this effect varies for each subject (Fig. 2f). Accordingly, killing activity by human neutrophils was impaired by oxalate and enhanced by TEPP-46 (Fig. 2g).

In addition to neutrophils, macrophages are also important phagocytic cells that produce ROS and contribute to bacterial killing. To verify whether our findings in neutrophils can also be extended to macrophages, we activated PKM2-deficient bone marrow-derived macrophages (BMDMs) with Zy/op (Supplementary Fig. 3a, b) or *S. aureus* (Supplementary Fig. 3c–e). Similar to neutrophils, PKM2 deficiency did not affect phagocytosis in BMDMs (Supplementary Fig. 3a, c). However, ROS production was actually slightly increased in BMDMs (Supplementary Fig. 3b, d). Despite this, there was no difference in the killing capacity of PKM2-deficient BMDMs (Supplementary Fig. 3e). Thus, PKM2 regulates ROS production and, consequently, killing activity in neutrophils but not in macrophages.

### Aerobic glycolysis is essential for ROS production in neutrophils

**Fig. 3:**
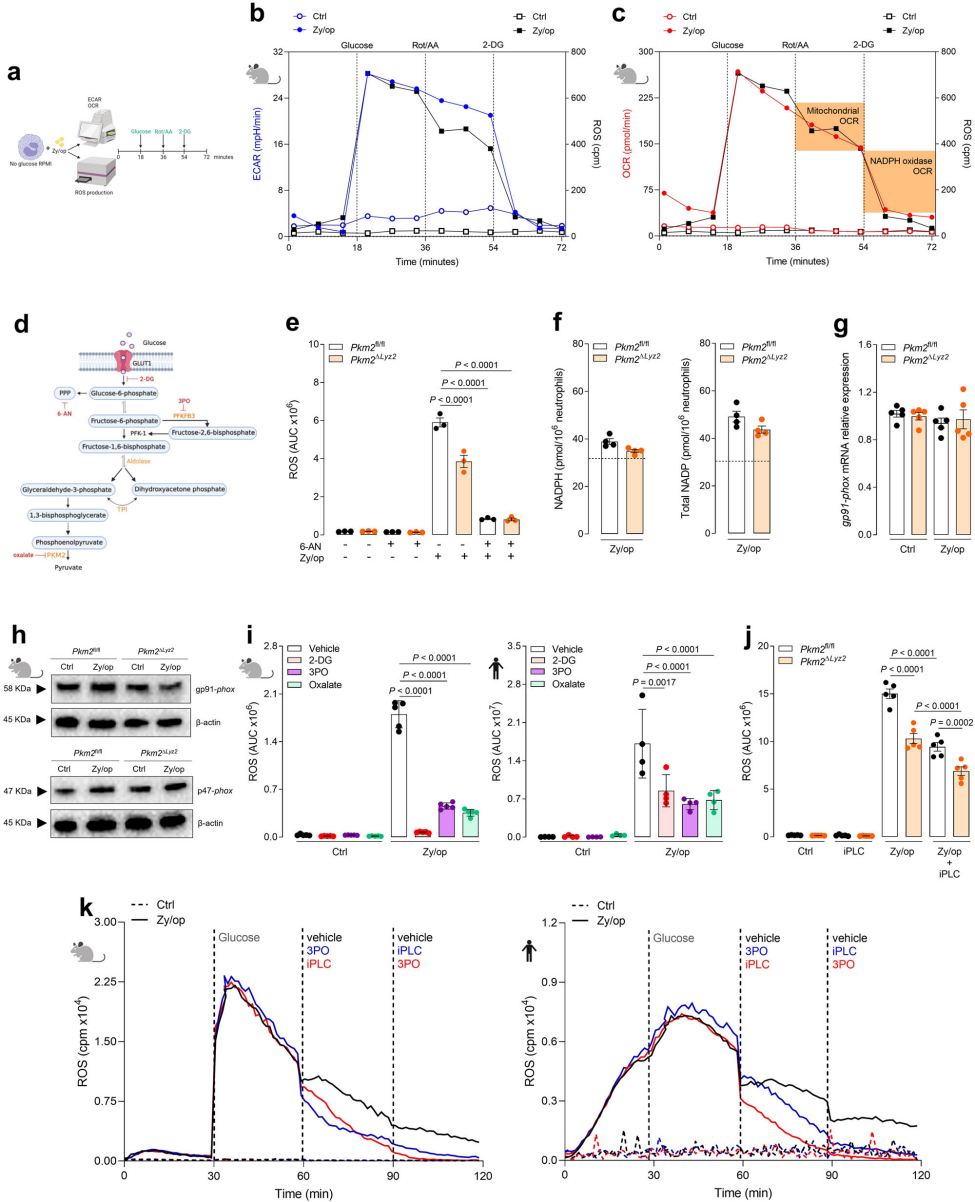
Aerobic glycolysis is essential for ROS production in activated neutrophils. **a** Schematic representation of the two simultaneous assays pooled together in the graphs of Fig. 3b, c. **b** Kinetic profile of ECAR and ROS and **c**, OCR and ROS production in neutrophils activated with Zy/op (100 µg/mL). *n* = 5 mice per group, representative of two independent experiments. **d** Schematic of the steps where the drugs inhibit glycolysis. **e** ROS production by neutrophils pre-treated with 6-AN (3 mM) for 1 h and then activated with Zy/op for 1 h. *n* = 3 mice per group, representative of three independent experiments. **f** NADPH and total NADP production by neutrophils activated with Zy/op for 30 min. *n* = 4 mice per group, representative of two independent experiments. **g** *gp91-phox* mRNA expression normalised to *Gapdh* in neutrophils activated with Zy/op for 3 h. *n* = 5 mice per group, representative of two independent experiments. **h** Immunoblot analysis of gp91-*phox* and p47-*phox* expression in neutrophils from wild-type and PKM2-deficient mice. β-actin was used as the loading control. Representative of two independent experiments. **i** ROS production by wild-type murine (left) or human (right) neutrophils pre-treated with 2-DG (3 mM), 3PO (10 µM), or oxalate (3 mM) for 1 h, then activated with Zy/op for 1 h. *n* = 5 mice per group in five independent experiments and *n* = 4 donors in four independent experiments, respectively. **j** ROS production by neutrophils pre-treated with a PLC inhibitor (iPLC, 1 µM) for 1 h, then activated with Zy/op for 1 h. *n* = 5 mice per group, representative of two independent experiments. **k** Representative real-time ROS production in wild-type and human neutrophils activated with Zy/op. ROS production was measured in response to glucose, 3PO, and iPLC at the indicated time points. *n* = 3 mice per group, representative of two independent experiments and *n* = 4 donors in four independent experiments, respectively. Error bars are mean ± SEM. *p* values were determined by two-tailed unpaired Student’s *t*-test (**f**) or one-way ANOVA followed by Tukey’s post hoc test (**b**, **c**, **e**, **g**, **i**–**k**). Clip art provided by Biorender. Source data are provided in the Source data file.

The NADPH oxidase complex catalyses superoxide production, the primary source of ROS in neutrophils, a process that requires a large amount of NADPH^2^. Among the different pathways contributing to NADPH production, the activation of PPP is crucial to trigger oxidative burst in neutrophils^9^. Since ROS production was impaired in murine and human neutrophils in the absence of glucose (Supplementary Fig. 4a), we next evaluated the relationship between glycolytic activity and ROS production. To this end, we performed a kinetic experiment using two simultaneous assays, ROS production by chemiluminescence and glycolysis stress test using the Seahorse XF96 Analyzer (Agilent Technologies), plotting the results in the same graph for comparison (Fig. 3a). Murine neutrophils in glucose-free media were stimulated with Zy/op just before starting the assays. For both assays, glucose was added at 18 min after initiation. At 36 min, rotenone and antimycin A (Rot/AA), complex I and complex III inhibitors, respectively, were added to shut down mitochondrial respiration; and, at 54 min, 2-DG was added to inhibit glycolysis. Figure 3b shows ECAR (extracellular acidification rate, blue line) and ROS production (black line). We found that Zy/op could not increase ECAR or ROS production without glucose. Interestingly, the addition of glucose increased ECAR, while Rot/AA had no effect, and 2-DG reduced ECAR to basal levels (blue line) (Fig. 3b). In parallel, we observed that ROS production increased when glucose was added with a slight decrease upon adding Rot/AA, but it was completely abolished when 2-DG was added. Together, these results show that glycolytic metabolism is crucial for ROS production by activated neutrophils. Since activated neutrophils increase oxygen consumption to produce ROS^22^, we next assessed the correlation between OCR (oxygen consumption rate), glucose metabolism, and ROS production (Fig. 3c). As shown in the red line, Zy/op was unable to increase OCR in the absence of glucose; however, OCR increased when glucose was added, slightly decreased when Rot/AA was added and returned to basal levels with the addition of 2-DG. Together with ROS results (black line), this highlights that while a portion of oxygen consumption and ROS production is due to mitochondrial respiration, both oxygen consumption and ROS production are dependent on glycolysis.

We then conducted a set of experiments targeting different steps of the glycolytic pathway to elucidate the importance of each one for regulating ROS production (Fig. 3d). First, wild-type and PKM2-deficient neutrophils were treated with the PPP inhibitor 6-AN (6-aminonicotinamide). We observed that while PKM2 deficiency partially reduced ROS production, 6-AN treatment resulted in a massive impairment in ROS production regardless of PKM2 presence (Fig. 3e), highlighting the well-known importance of PPP for this function^9^. To further explore whether PKM2-deficiency could impair PPP activation, thus leading to a decreased ROS production, we evaluated the NADPH and NADP concentrations in Zy/op-activated neutrophils, but no differences were found (Fig. 3f). We then examined the expression of some NADPH oxidase subunits. There were no differences in the gene expression of *gp91-phox* or protein expression of gp91-*phox* and p47-*phox* between wild-type and PKM2-deficient neutrophils (Fig. 3g, h). It has been shown that activated neutrophils have increased glutathione turnover, which may be consumed when ROS is produced^23^. Glutathione is an antioxidant molecule present at high concentrations in neutrophils, and the ratio between its reduced (GSH) and oxidised (GSSG) forms can be used to measure intracellular oxidative balance. However, PKM2 deficiency did not alter the concentration of total glutathione or GSH/GSSG ratio in neutrophils (Supplementary Fig. 4b). Second, we treated murine and human neutrophils with 2-DG to inhibit hexokinase (HK), 3PO to inhibit PFKFB3 and oxalate to inhibit PK, at doses that showed comparable inhibition of lactate production. As shown in Fig. 3i, the inhibition of glycolysis at HK, PFKFB3, or PK decreased ROS production. Altogether these results show that different glycolytic steps are important for ROS production; however, PKM2 regulates ROS production by a PPP-independent mechanism.

Besides being dependent on glycolysis, ROS production also depends on PLC activation, which results in calcium release, DAG production, and, consequently, PKC activation^24^. We performed two experiments to better understand the role of the PLC-dependent and the glucose-dependent pathways in ROS production. In the first one, wild-type and PKM2-deficient neutrophils were pre-treated with a PLC inhibitor (iPLC) for 1 h before being stimulated with Zy/op. PLC inhibition decreased ROS production in both cells, but it had an additive effect in PKM2-deficient neutrophils (Fig. 3j), suggesting that PKM2 regulates ROS production independent of PLC. In the second one, murine or human neutrophils were activated with Zy/op without glucose (Fig. 3k). After 30 min, glucose was added to the media, and an increase in ROS production was observed. After 30 min, we added vehicle (black line), 3PO (blue line), or iPLC (red line) and found that both inhibitors decreased ROS production, showing that both pathways are important for this function. Finally, after 30 min, we added vehicle (black line), iPLC (blue line) or 3PO (red line) and found that, in both cases, ROS production decreased to basal levels, showing that PLC-dependent and glucose-dependent pathways complement each other for ROS production. Together, these results suggest that PKM2-mediated glycolysis and PLC pathway are independently required for ROS production in activated neutrophils.

### The metabolic activity of PKM2 regulates ROS production in neutrophils

**Fig. 4:**
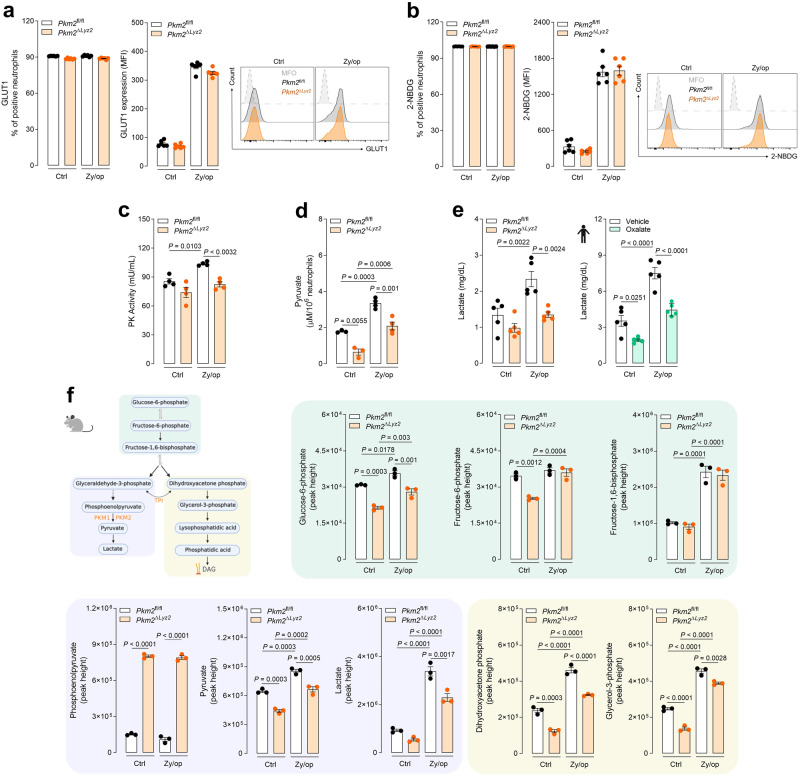
Metabolic activity of PKM2 regulates ROS production in neutrophils. **a** GLUT1 expression in wild-type (*Pkm2*^fl/fl^) and PKM2-deficient (*Pkm2*^∆*Lyz2*^) activated with Zy/op (100 µg/mL) for 1 h. Representative histogram and graph bar showing the GLUT1 frequency and median fluorescence intensity (MFI). Gating strategies are shown in Supplementary Fig. 5a. *n* = 6 mice per group in two independent experiments. **b** Glucose uptake in wild-type and PKM2-deficient neutrophils activated with Zy/op for 30 min. Representative histogram and graph bar showing frequency and MFI for 2-NBDG. Gating strategies are shown in Supplementary Fig. 5a. *n* = 6 mice per group in two independent experiments. **c** Pyruvate Kinase (PK) activity in neutrophils activated with Zy/op for 1 h. *n* = 5 mice per group in two independent experiments. **d** Pyruvate production by neutrophils activated with Zy/op for 1 h. *n* = 4 mice per group in two independent experiments. **e** Lactate production by wild-type and PKM2-deficient mouse neutrophils and by human neutrophils pre-treated with oxalate (3 mM) for 1 h and activated with Zy/op for 1 h. *n* = 5 mice per group in two independent experiments and *n* = 5 donors in five independent experiments. **f** Glycolytic metabolites from ^13^C-glucose tracing in wild-type and PKM2-deficient neutrophils activated with Zy/op for 1 h. The *y*-axis refers to the height of the chromatography peak obtained by LC-MS. *n* = 3 mice per group in three independent experiments. Error bars are mean ± SEM. *p* values were determined by one-way ANOVA followed by Tukey’s post hoc test. Clip art provided by Biorender. Source data are provided in the Source data file.

Because PKM2, acting as a transcriptional coactivator, has been reported to have an important role in the expression of glycolytic genes and induction of glycolytic flux^25^, we next investigated whether PKM2 deficiency affects the expression of glucose transporter 1 (GLUT1) or glucose uptake (evaluated by flow cytometer analysis using 2-NBDG) in activated neutrophils. As expected, we found an upregulation of GLUT1 expression (Fig. 4a) and glucose uptake (Fig. 4b) in Zy/op-activated neutrophils compared to control neutrophils. However, PKM2 deficiency did  not affect these parameters (Fig. 4a, b). We then evaluated total pyruvate kinase activity (including PKM1 and PKM2) and found a significant reduction in PKM2-deficient neutrophils activated with Zy/op (Fig. 4c). In accordance, Zy/op-activated control neutrophils showed increased pyruvate (Fig. 4d) and lactate (Fig. 4e) production when compared to PKM2-deficient neutrophils. In line, oxalate-treated human neutrophils showed a decreased lactate production (Fig. 4e). Despite mitochondrial ROS production being increased in PKM2-deficient neutrophils (Supplementary Fig. 5a), no mitochondrial dysfunction was observed in these cells, as evaluated by MitoTracker Green and Red (Supplementary Fig. 5b). Taken together, these results show that PKM2 is required for the canonical metabolic function (pyruvate kinase activity) rather than transcriptional control activity in activated neutrophils.

To further explore the metabolic changes regulated by PKM2, we measured the redistribution of glucose metabolism flux during neutrophil activation by a tracing study with ^13^C-glucose. Zy/op-activated wild-type neutrophils showed an increase in glycolysis metabolites, including G6P, F1,6BP, pyruvate, and lactate, consistent with the activation of the glycolytic pathway. Importantly, DHAP and G3P, the precursors for DAG de novo synthesis from glucose, were also increased (Fig. 4f). We then evaluated the glucose metabolism flux in PKM2-deficient neutrophils and found that, as in wild-type neutrophils, F1,6BP production was also increased in PKM2-deficient neutrophils activated with Zy/op. However, pyruvate and lactate, in addition to DHAP and G3P, were all decreased (Fig. 4f and Supplementary Fig. 6a, b). Importantly, PEP was hugely accumulated in PKM2-deficient neutrophils. To better understand the direction of the glycolytic flux in neutrophils (i.e., whether it favours lactate or DAG production), we evaluated the GAP and DHAP concentrations in Zy/op-activated murine neutrophils. Both metabolites were boosted, confirming that both pathways were activated during neutrophil activation (Supplementary Fig. 6c, d). In addition, we found that GAP accumulates, and the production of DHAP is impaired when PKM2 is inhibited by oxalate (Supplementary Fig. 6c, d). These data indicate that PKM2 activity regulates the DHAP pathway leading to de novo synthesis of DAG from glucose.

### Phosphoenolpyruvate accumulation reduces ROS production in neutrophils

**Fig. 5:**
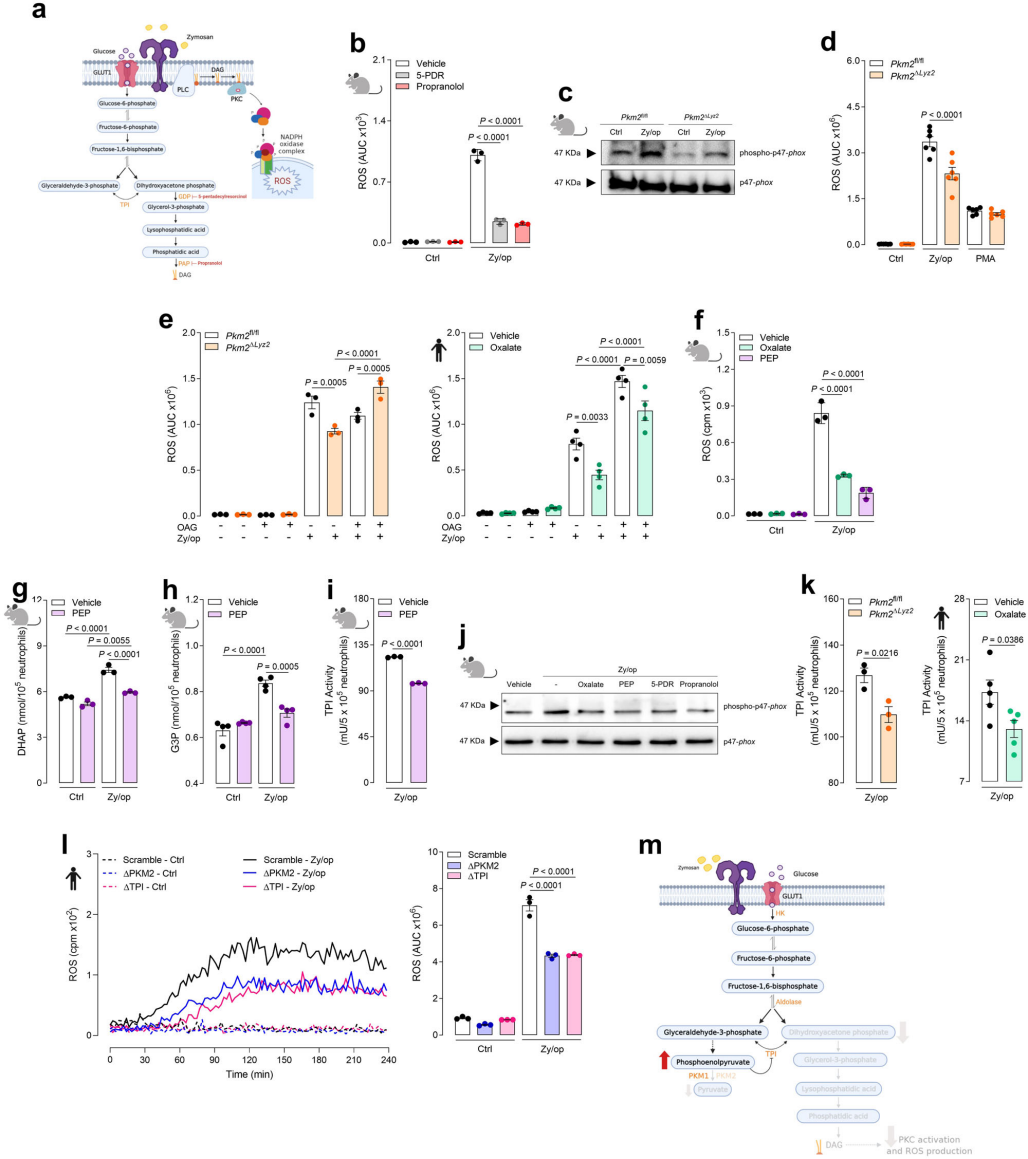
PKM2 modulates ROS production in neutrophils by a TPI-dependent mechanism. **a** Schematic of glycolysis inhibition by various drugs. **b** ROS production by neutrophils treated with 5-PDR (30 µM) or propranolol (30 µM) for 1 h and activated with Zy/op for 1 h. *n* = 3 mice per group, representative of three independent experiments. **c** Immunoblot analysis of p47-*phox* phosphorylation in wild-type (*Pkm2*^fl/fl^) and PKM2-deficient (*Pkm2*^∆*Lyz2*^) neutrophils activated with Zy/op for 30 min. Representative of two independent experiments. **d** ROS production by neutrophils activated with Zy/op or PMA (50 nM) for 1 h. *n* = 6 mice per group, representative of three independent experiments. **e** ROS production by murine and human neutrophils pre-treated with oxalate (3 mM) for 1 h and activated with Zy/op for 1 h in the presence of OAG (10 µM). *n* = 3 mice per group in three independent experiments and *n* = 4 donors in four independent experiments, respectively. **f** ROS production by neutrophils pre-treated with oxalate or PEP (1 mM) for 1 h and activated with Zy/op for 1 h. *n* = 3 mice per group, representative of three independent experiments. **g** DHAP production by neutrophils pre-treated with PEP for 1 h and activated with Zy/op for 1 h. *n* = 3 mice per group, representative of two independent experiments. **h** G3P production by neutrophils pre-treated with PEP and activated with Zy/op. *n* = 4 mice per group, representative of two independent experiments. **i** TPI activity in neutrophils pre-treated with PEP and activated with Zy/op. *n* = 3 mice per group, representative of two independent experiments. **j** Immunoblot analysis of p47-*phox* phosphorylation in wild-type neutrophils pre-treated with oxalate, PEP, 5-PDR or propranolol for 1 h and activated with Zy/op for 30 min. Representative of two independent experiments. **k** TPI activity in murine and in human neutrophils pre-treated with oxalate for 1 h and activated with Zy/op for 1 h. *n* = 3 mice per group (representative of two independent experiments) and *n* = 5 donors (representative of five pooled independent experiments). **l** ROS production by scramble, PKM2-(∆PKM2) or TPI-(∆TPI) deficient NB4 cells activated with Zy/op for 1 h. *n* = 3 independent experiments. **m** Working hypothesis. Error bars are mean ± SEM. *p* values were determined by two-tailed unpaired Student’s *t*-test (**I**, **k**) or one-way ANOVA followed by Tukey’s post hoc test (**b**, **d**–**h**, **l**). Clip art provided by Biorender. Source data are provided in the Source data file.

To elucidate whether the DHAP pathway that leads to DAG production from glucose is important for ROS production, we pre-treated murine neutrophils with 5-pentadecylresorcinol (5-PDR), an inhibitor of glycerol-3-phosphate dehydrogenase (GDP), or propranolol, an inhibitor of phosphatidate phosphohydrolase (Fig. 5a). We found that both inhibitors impaired ROS production in Zy/op-activated neutrophils (Fig. 5b). These results, together with the glucose tracing analysis, indicate that PKM2 deficiency or inhibition could decrease ROS production by downregulating DHAP and G3P production and, consequently, DAG synthesis from glucose, thus decreasing NADPH oxidase complex activation. In fact, PKM2-deficient neutrophils activated with Zy/op had reduced phosphorylation of p47-*phox*, indicating an impaired activation of the NADPH oxidase complex (Fig. 5c). In addition, neutrophil activation with PMA — a PKC direct activator — bypassed the PKM2 deficiency role in ROS production (Fig. 5d), showing that the impairment in ROS production in PKM2-deficient neutrophils is due to an upstream event for PKC activation.

To further explore the importance of DAG on ROS production, we examined whether 1-oleoyl-2-acetyl-sn-glycerol (OAG), a synthetic DAG analogue, would rescue ROS production in PKM2-deficient neutrophils or oxalate-treated human neutrophils. We found that OAG restored ROS production Zy/op-activated neutrophils in both cases (Fig. 5e). Together, these data indicate that PKM2 regulates ROS production in activated neutrophils by modulating DAG de novo synthesis from glucose. In this way, two independent pathways — through PLC activation and de novo synthesis from glucose — are responsible for maintaining DAG levels in neutrophils, which is important for PKC and NADPH oxidase activation.

Grüning and collaborators^26^ showed that a decrease in pyruvate kinase activity leads to PEP accumulation in yeast, inhibiting the enzyme TPI by binding to its catalytic pocket. Accordingly, we also found that PEP accumulates in PKM2-deficient neutrophils (Fig. 4f). TPI interconverts GAP and DHAP^27^, and PEP competes with GAP for binding to TPI^26^. We then hypothesised that PEP accumulates and inhibits TPI activity when PKM2 is absent or inhibited in neutrophils. This would decrease DHAP accumulation and, consequently, DAG production, impairing ROS production. To further explore this hypothesis, we treated wild-type neutrophils with PEP for 1 h prior to the activation with Zy/op and found that ROS production was impaired (Fig. 5f). In addition, DHAP (Fig. 5g) and G3P (Fig. 5h) concentrations and TPI activity (Fig. 5i) were also reduced. Furthermore, PEP and oxalate treatment decreased phosphorylation of p47-*phox*, which indicates a decreased NADPH oxidase complex activation (Fig. 5j). We then evaluated TPI activity in PKM2-deficient neutrophils and oxalate-treated human neutrophils. We found that the absence or inhibition of PKM2 decreased TPI activity (Fig. 5k). To further assess TPI importance for ROS production, we utilised CRISPR/Cas9-based editing to disrupt *Pkm2* and *Tpi* (Supplementary Fig. 7a–c) in NB4 cells, a promyelocytic leukaemia cell line that differentiates to maturate neutrophils upon exposure to all-trans retinoic acid (ATRA)^28^. We found that ROS production was impaired in the absence of PKM2 and TPI (Fig. 5l). Together, these results show that PKM2 deficiency reduces ROS production in activated neutrophils by increasing PEP accumulation, which inhibits TPI activity and DHAP metabolism, impairing NADPH oxidase activation and ROS production (Fig. 5m). In this way, PKM2 regulates the DHAP-DAG-PKC-NADPH oxidase axis to promote ROS production in neutrophils.

### PKM2 is important for *S. aureus* killing in vivo

**Fig. 6:**
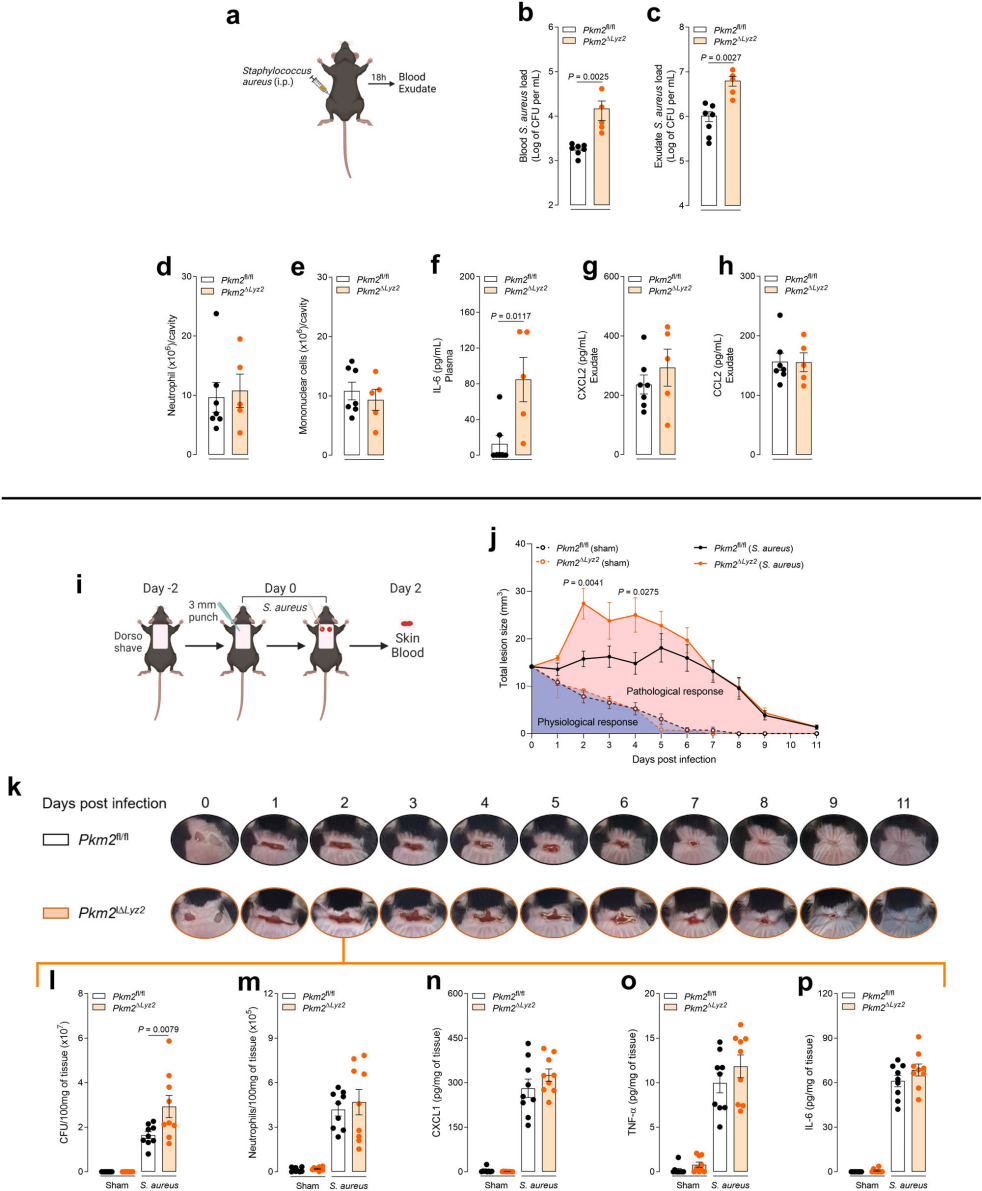
PKM2-deficiency in myeloid cells impairs host defence against *S. aureus* infection. **a** Schematic protocol of peritonitis infection with *S. aureus*. **b** Bacterial load in the blood 18 h after challenge in *Pkm2*^fl/fl^ and *Pkm2*^∆*Lyz2*^ mice. *n* = 7 and 5 mice, respectively. Representative of two independent experiments. **c** Bacterial load in the exudate 18 h after challenge in *Pkm2*^fl/fl^ and *Pkm2*^∆*Lyz2*^ mice. *n* = 7 and 5 mice, respectively. Representative of two independent experiments. **d** Neutrophil cell counts 18 h after challenge in *Pkm2*^fl/fl^ and *Pkm2*^∆*Lyz2*^ mice. *n* = 7 and 5 mice, respectively. Representative of two independent experiments. **e** Mononuclear cell counts 18 h after challenge in *Pkm2*^fl/fl^ and *Pkm2*^∆*Lyz2*^ mice. *n* = 7 and 5 mice, respectively. Representative of two independent experiments. **f**–**h** Concentration of IL-6, CXCL2, and CCL2 in the plasma and exudate 18 h after *S. aureus* challenge in *Pkm2*^fl/fl^ and *Pkm2*^∆*Lyz2*^ mice. *n* = 7 and 5 mice, respectively. Representative of two independent experiments. **i** Schematic protocol of localised cutaneous infection with *S. aureus*. **j** Mean of total lesion size (cm^2^) ± the standard error of the mean (SEM) evaluated for 11 days. *n* = 10 mice. Representative of two independent experiments. **k** Representative pictures of the dorsal skin lesions in the back of *Pkm2*^fl/fl^ and *Pkm2*^∆*Lyz2*^ mice evaluated for 11 days. **l** Bacterial load in the wound two days after challenge in *Pkm2*^fl/fl^ and *Pkm2*^∆*Lyz2*^ mice. *n* = 9 mice. Representative of two independent experiments. **m** Myeloperoxidase activity evaluated in the wound two days after *S. aureus* challenge in *Pkm2*^fl/fl^ and *Pkm2*^∆*Lyz2*^ mice. *n* = 9 mice. Representative of two independent experiments. **n**–**p** Concentration of CXCL1, TNF-α and IL-6 in the wound two days after *S. aureus* challenge in *Pkm2*^fl/fl^ and *Pkm2*^∆*Lyz2*^ mice. *n* = 9 mice. Representative of two independent experiments. Error bars are mean ± SEM. *p* values were determined by one-way ANOVA followed by Tukey’s post hoc test. Clip art provided by Biorender. Source data are provided in the Source data file.

*S. aureus* is a Gram-positive bacterium that is a common pathogen responsible for a skin infection^29^. A hallmark of *S. aureus* infections is neutrophil recruitment and abscess formation, which is required to eliminate the pathogen^30,31^. To determine the importance of PKM2 in vivo, we used two different mouse models: peritonitis and cutaneous infection with *S. aureus*^32^ in *Pkm2*^fl/fl^ and *Pkm2*^∆*Lyz2*^ mice and evaluated the infection progression. For the first model, we inoculated mice with *S. aureus* via intraperitoneal (i.p.) injection, and the animals were assessed for 18 h (Fig. 6a). We found that *Pkm2*^∆*Lyz2*^ mice were more susceptible to *S. aureus* infection than *Pkm2*^fl/fl^ mice, showing higher bacterial loads in the blood (Fig. 6b) and in the peritoneal exudate (Fig. 6c). Despite that, neutrophils (Fig. 6d) and mononuclear cells (Fig. 6e) counts were unchanged. In addition, IL-6 was increased in the plasma (Fig. 6f) of *Pkm2*^∆*Lyz2*^ mice, but no differences were found in CXCL2 (Fig. 6g) and CCL2 (Fig. 6h) levels in the exudate.

It has been shown that mice inoculated intracutaneously with high doses of *S. aureus* display clinical and histopathological signs of local infection and inflammation and massive neutrophil recruitment within 48 h^33^. In this way, for the second model, we performed two 3 mm punches through the dermis in the back of shaved mice and inoculated the wound with *S. aureus* (Fig. 6i). The wound lesion sizes of anaesthetised mice were evaluated for 11 days and determined by measuring the total lesion size (square centimeters). We found that sham *Pkm2*^fl/fl^ and *Pkm2*^∆*Lyz2*^ mice had the same wound healing timing, showing no difference in the cicatrisation processes in mice that do not express PKM2 in myeloid cells (Fig. 6f). However, when inoculated with *S. aureus*, *Pkm2*^∆*Lyz2*^ mice had larger wounds than *Pkm2*^fl/fl^ mice in the first four days of infection (Fig. 6j, k). Moreover, compared to *Pkm2*^fl/fl^, the wound from *Pkm2*^∆*Lyz2*^ had more colony-forming units (CFU) of *S. aureus* at 48 h of inoculation despite having the same number of neutrophils at later time points (Fig. 6l, m). In addition, there was no difference in the concentrations of inflammatory cytokines CXCL1 (Fig. 6n), TNF-α (Fig. 6o), and IL-6 (Fig. 6p) in the wound tissue homogenates. Altogether these results show that PKM2 is important for controlling *S. aureus* infection in vivo.

## Discussion

In the past few years, the role of metabolic reprogramming as a key immuno-regulatory phenomenon has been investigated by its influence on cell differentiation, function, and fate^34^. In this context, the enzyme PKM2 has been implicated as a critical regulator of aerobic glycolysis and recently has generated significant interest due to its upregulation in activated immune cells^15^. Of note, it was shown that in macrophages and lymphocytes, PKM2 has a major transcriptional role, migrating to the nucleus and regulating gene expression^16–18^. Conversely, we show here that PKM2 is mainly expressed in the cytoplasm of neutrophils. Its expression in the tetrameric form is upregulated during neutrophil activation, indicating that PKM2’s primary role is on the glycolytic pathway in activated neutrophils.

Neutrophils are rapidly recruited to tissues to phagocytose and eliminate invading pathogens via antimicrobial actions, especially ROS production^2,35^. It is worth noting that neutrophils recognise both Zymosan and *S. aureus* by TLR2, which bind to ligands with very diverse structures such as lipoproteins/lipopeptides, peptidoglycan, glycopolymers, and proteins^36^. Here, we showed that while PKM2 was not important for the phagocytosis process, it regulates ROS production and, consequently, the killing capacity in neutrophils. It is already known that ROS production is dependent on glycolysis^7^, but until now, this was mainly associated with the PPP activation and the balance of NADPH/NADP in neutrophils^6,10,37^. Surprisingly, we found that, in the absence of PKM2, ROS production was reduced without impairments in NADPH/NADP balance, while PK activity and lactate production was decreased.

We found a difference between murine and human neutrophil responses during our experiments utilising glucose-free media. The absence of glucose almost totally impaired ROS production in murine neutrophils and reduced it by 50% in human neutrophils. In this regard, it is interesting to note that neutrophils contain glycogen within granules, which is important to their function and survival. Furthermore, human neutrophils contain more glycogen than murine neutrophils^38^, which could explain the more drastic effect of glucose starvation in ROS production of murine neutrophils. Moreover, we found that ROS production is associated with increased glycolytic rate and the inhibition of glycolysis in different steps of this pathway impaired ROS production. Previously, the canonical pathway for ROS production was not thought to involve the direct activation of the glycolytic pathway, and it has been shown that glycolysis provides the NADPH used during NADPH oxidase activation for ROS production^39^. However, our results highlight that PEP, a glycolytic metabolite at PKM2 enzymatic reaction, is involved in regulating ROS production in activated neutrophils.

DAG is an important PKC activator and can be produced from glucose in neutrophils in a pathway dependent on DHAP metabolism^11^. However, if DAG produced from glucose/DHAP metabolism contributes to ROS production is unknown. We found that the inhibition of DAG production from glycolysis by blocking the glycerol-3-phosphate dehydrogenase, which converts DHAP into G3P, and the phosphatidate phosphohydrolase, which converts phosphatidic acid into DAG, impaired ROS production. In addition, as demonstrated by others^38^, we found here that neutrophil activation increased DHAP production. However, DHAP and G3P concentrations were decreased in PKM2-deficient and oxalate-treated neutrophils, indicating that PKM2 somehow regulates the DHAP downstream pathway. As aforementioned, this pathway is important for de novo synthesis of DAG. In fact, the treatment of neutrophils with OAG restored ROS production in PKM2-deficient and oxalate-treated neutrophils, suggesting that ROS production is impaired in these cells because DAG production from glucose is decreased.

It has been shown that a decrease of pyruvate kinase activity leads to PEP accumulation, which can inhibit the activity of triose phosphate isomerase (TPI) by binding to its catalytic pocket^26^. TPI has high catalytic efficiency and enhances the movement of a single proton to constantly interconvert DHAP and GAP, where the equilibrium favours the synthesis of DHAP^40,41^. Here we found a decrease in TPI activity in PKM2-deficient and oxalate-treated neutrophils. We hypothesised that PKM2 modulates TPI activity by regulating PEP concentration, which would impact on DHAP concentration and, ultimately, ROS production, explaining, at least in part, our results. Indeed, when neutrophils were treated with PEP, there was a decrease in ROS production, DHAP, and G3P concentrations. Conversely, it has been shown that the low TPI activity increases DHAP concentration in erythrocytes^42^ and yeast^26^ and mediates an increase in PPP metabolites concentrations and oxidant resistance in yeast^26^. However, PKM2-deficient neutrophils had normal glutathione concentrations, suggesting that, in contrast to what is seen in other cell types, the low TPI activity did not alter the PPP activation in neutrophils. Thus, further studies are needed to understand the role of TPI in neutrophil function.

Finally, we showed that PKM2 is important for early infection resolution in vivo in two different *S. aureus* infection models, peritonitis and skin infection, where a massive neutrophil infiltration is observed in the first hours of infection^43^. The importance of glycolysis for *S. aureus* killing by neutrophils was shown by Boxer and collaborators^5^, where neutrophils treated with 2-DG had impaired killing capacity. Furthermore, Burge and collaborators^44^ showed that a patient with recurrent *S. aureus* infections had a defect in PK activity. In both models we evaluated, there was a higher number of *S. aureus* CFU despite having the same number of neutrophils in the peritoneum and the wound. It is known that defects in neutrophil functions, including ROS production, chemotaxis, and granules disorders impair *S. aureus* elimination and infection resolution^43^. Together with our in vitro findings, these results indicate that PKM2 regulation of ROS production in neutrophils is important for bacterial elimination in vivo.

However, because we used *Lyz2*^cre^ PKM2^fl/fl^ mice, we cannot exclude that PKM2 plays additional roles in macrophages that could account for controlling *S. aureus* infection in vivo.

Our results reveal a new role of PKM2 that regulates the DHAP-DAG-PKC-NADPH oxidase axis and, at least in part, ROS production in neutrophils by impairing PEP accumulation. These findings fill the gaps in the current knowledge of the importance of the glycolytic pathway for ROS production and provide insight into the mechanisms underlying the role of PKM2 in neutrophil metabolism and function.

## Methods

### Animals

C57BL/6 wild-type mice were purchased from Charles River. *Lyz2*^Cre^ (strain# 004781) and *PKM2*^flox/flox^ (strain# 024048) mice were obtained from Jackson Laboratories. Myeloid cell-(*Pkm2*^Δ*Lyz2*^) specific-*Pkm2*-deficient mice were generated by crossing the *Pkm2*^flox/flox^ mice with *Lyz2*^Cre^ mice on a C57BL/6 background. *Pkm2*^fl/fl^ mice without *Lyz2*^Cre^ gene were used as controls for all experiments. All experiments were carried out with 6–7-week-old female and male littermate mice. The protocols used for animal experimentation were approved by the Animal Welfare Committee of the Ribeirão Preto Medical School, University of São Paulo (protocol number: 143/2017). Animals were bred and maintained under specific pathogen-free conditions (12/12-h light/dark cycle, 55 ± 5% humidity, 21–23 °C) at the animal facility of the Ribeirão Preto Medical School, University of São Paulo. Mice were randomly assigned to experimental groups. Mice were euthanized with Xylazine/Ketamine (10 mg/kg / 80 mg/kg) administrated intraperitoneally (i.p.).

### Human blood samples

Thirty healthy donors of both sexes with ages between 18 and 40 years were included in this study. All recruited volunteers provided written informed consent form. The exclusion criteria were smoking, obesity, diabetes, pregnancy and anti-inflammatory use. The study was approved by the Human Subjects Institutional Committee of the Ribeirão Preto Medical School, Brazil (License number: 31033519.4.0000.5440).

### Neutrophil isolation and culture

Murine neutrophils were obtained from mouse bone marrow. Briefly, mouse femurs and tibias were removed and flushed with phosphate buffer solution (PBS, Corning) and then isolated by immunomagnetic negative selection with the Neutrophil Isolation Kit, following manufacturer’s instructions (Miltenyi Biotech, 130-097-658).

Human neutrophils were obtained from peripheral blood by Percoll (GE Healthcare, 17-0891-01) density gradient^45^. Briefly, whole blood was collected into Vacutainer® Citrate Tubes with 3.2% buffered sodium citrate solution as anticoagulant (BD Bioscience). Blood was laid on top of a 4-layer Percoll gradient (72, 63, 54 and 45% in PBS) and then centrifuged at 650 × *g*, 30 min, at 20 °C. Neutrophils were recovered at the interface of the 63–72% fractions. The remaining erythrocytes were lysed (1X RBC Lysis Buffer, Thermo, 00-4333-57).

Both murine and human neutrophils were resuspended in Hank’s balanced saline solution (HBSS, Corning, 21-020-CM) containing 2% of bovine serum albumin (BSA) and calcium. Cell suspensions contained >95% of viable neutrophils, as established by flow cytometry.

### Bone marrow-derived macrophages (BMDM) isolation and culture

BMDMs were obtained using L929 cell-conditioned medium, as previously described^46^. Briefly, bone marrow cells were obtained by flushing both mice femurs and tibias with RPMI-1640 culture media (Corning, 15-040-CV). After centrifugation, cells were resuspended in RPMI-1640 containing 20% L929, 10% foetal bovine serum (FBS, GE Life Sciences, SV30160.03), L-glutamine (2 mM, Sigma, G7513), penicillin (100 U/mL, Sigma, P4333), and fungizone (2.5 μg/mL, Gibco, 15-290-018) and seeded for 7 days. After the end of this period, BMDMs were harvested for subsequent experiments.

### CRISPR/Cas9 gene editing in NB4 cell line

NB4 cell line (ATCC, ACC-207) was cultured in RPMI-1640 culture medium supplemented with 10% FBS, penicillin (100 U/ml), and L-glutamine (2 mM) at 37 °C and 5% CO_2_. For the generation of NB4 deficient for PKM2 and TPI, we used the CRISPR/Cas9 plasmid PXL (plasmid # 75349, Addgene). For the selection of the gene region to be targeted by the Cas9 endonuclease, we utilised Benchiling (Biology Software), selecting the exon 10 of the coding sequence of the PKM gene or exon 3 of the coding sequence of TPI, all responsible for the production of their respective protein. Succinctly, based on those sequences, guide RNAs were selected using CRISPOR^47^. The oligonucleotides synthesised carrying the targeting sequence of PKM2 were 5′-caccATTTGAGGAACTCCGCCGCC-3′ and 5′- aaacGGCGGCGGAGTTCCTCAAAT−3′. For TPI 5′-acaccGCAAAGACTGCGGAGCCACGTG-3′ and 5′-aaaacACGTGGCTCCGCAGTCTTTGCG-3′ (Exxtend). The oligos were then cloned into the PXL vector (Addgene, 75349)^48^. Briefly, *E. coli* DH5-α chemo-competent bacteria were transformed with the plasmid, and on the next day, the plasmids were purified by the method of MiniPrep, following the manufacturer’s instructions (Qiagen-Hilden, 27104). NB4 cells (2 × 10^6^ cells) were resuspended in 100 μl of Cell Line Nucleofector Solution V buffer. To this solution was added 2 μg of the cloned PXL plasmid. This mixture was transferred to cuvettes, and electroporation was performed using the X-001 (Nucleofector I Device) protocol. After transfection, 500 μl of RPMI-1640 supplemented with 10% FBS was immediately added, and the cells were plated. After 48 h, NB4 cells were differentiated into granulocytes with 1 µM ATRA (Sigma-Aldrich) for 5 days. Granulocytic differentiation was evaluated by cell morphology.

### Mouse model of skin wound infection

The mouse model of skin wound infection was done as described by Guo and collaborators^33^, with modifications. Briefly, mice were anesthetised by inhalation administration of isoflurane (1–3%), and their posterior backs were shaved. On the following day, two full-thickness wounds were made through the dermis using a 3 mm biopsy punch. The wounds were subsequently inoculated with *Staphylococcus aureus* (ATCC, Strain 6538, 1 × 10^8^ CFU per 7.5 µl of PBS in each wound) using a micropipette. Wounds of sham mice were not inoculated. All mice were given analgesics (12.5 mg.kg^−1^, tramadol, Agener União, subcutaneous) beginning 30 min before making the wounds and then every 12 h up to day 3. Measurements of total lesion size (square centimetres) were made daily.

Wound healing was evaluated for 11 days. For wound analysis, biopsy skin specimens were obtained from the lesions of infected mice. Biopsy of healthy skin from the same mouse was used as a control. Tissues were weighed before being homogenised in 500 µL PBS. Recovered bacteria were counted by plating serial dilutions onto Mueller-Hinton agar (Difco, 225250) plates. Samples were centrifuged, and supernatants were used for cytokines measurements. The pellet was used for MPO activity assay by measuring the change in OD at 450 nm using tetramethylbenzidine^49^. This assay was used as a quantitative measurement of neutrophil migration and accumulation in the wound.

### Mouse model of peritonitis infection

Mice were anesthetised by inhalation administration of isoflurane (1–3%) and inoculated with *S. aureus* (1 × 10^8^ CFU) intraperitoneally (i.p.). After 18 h, mice were euthanised, and exudate and blood were obtained. Recovered bacteria were counted by plating serial dilutions onto Mueller-Hinton agar plates. Samples were centrifuged and supernatants were used for cytokines measurements.

### Zymosan opsonisation

For Zymosan opsonisation, 10 mg of zymosan (Sigma, Z4250) was washed in PBS and then incubated with murine or human serum diluted twice in PBS at 37 °C for 30 min. Zymosan opsonised (Zy/op) was washed twice, resuspended in PBS and stored at −20 °C. For phagocytosis experiments, Zymosan was first labelled with Fluorescein isothiocyanate isomer I (FITC, Sigma, F4274) and then incubated with serum (Zy-FITC/op).

### Bacteria

*Staphylococcus aureus* was obtained from the American Type Culture Collection (ATCC, USA, Strain 6538). Bacteria were cultivated from frozen stocks for 24 h at 37 °C in Brain Heart Infusion Agar (Oxoid, CM1135). Bacteria suspension was prepared in sterile NaCl 0.9% and the calculations were estimated by measuring the OD of solutions at 600 nm. CFU were verified by plating serial dilutions onto Mueller-Hinton agar plates.

### Western blotting

For standard western blot protocols, neutrophils (3 × 10^6^) were activated with Zy/op (100 µg/mL) at 37 °C and 5% CO_2_. After 6 h, cells were harvested in boiling Laemmli Sample Buffer (Biorad, 161-0737), followed by heating at 95 °C for 5 min. For crosslink assay, neutrophils were incubated with 500 µM disuccinimidyl suberate (Sigma-Aldrich, S1885) for 30 min, and then cell lysates were prepared as described above. Protein samples were resolved on SDS-PAGE gels and transferred onto a nitrocellulose membrane (GE Healthcare). Membranes were blocked with 5% (wt/vol) non-fat milk (Cell Signalling) in Tris-buffered saline with 0.1% Tween-20 (TBST) for 1 h at room temperature and then incubated overnight at 4 °C with 1:1000 dilutions of primary antibodies against PKM2 (Cell signalling, Cat# 4053 P), β-actin (Cell Signalling, Cat# 3700 S), gp91-*phox* (BD Transductions, Cat# 611414), p47-*phox* (Millipore, Cat# 07-500) and phospho-p47-*phox* (Invitrogen, Cat# PA5-36863). Membranes were repeatedly washed with TBST and incubated for 2 h with the appropriate HRP-conjugated secondary antibody (1:5000 dilution; Sigma-Aldrich, anti-rabbit Cat# A0545; anti-mouse Cat# A9044). Immunoreactivity was detected using the ECL prime reagent (GE Healthcare, RPN2236), and then the chemiluminescence signal was recorded on the ChemiDoc XRS Imager (Bio-Rad Laboratories). Data were analysed with Image Lab software (Bio-Rad Laboratories). Total β-actin levels were used as a loading control.

### Confocal microscopy

Neutrophils (8 × 10^4^) were incubated on poly-L-lysine–coated coverslips for 30 min and activated with Zy/op (100 µg/mL) for 6 h. After this time cells were fixed (2% PFA), permeabilised and stained with anti-PKM2 (1:200, Cell signalling, Cat# 4053P) overnight. After incubation with a secondary antibody (1:800, AlexaFluor 488, Abcam, Cat# ab150065), coverslips were washed in PBS and mounted onto microscope slides using a DAPI-containing mounting medium (Vector Laboratories, H-1200-10). Images were acquired by Axio Observer combined with LSM 800 microscope (Carl Zeiss) and DMI6000B microscope (Leica Microsystems) and analysed with Fiji/ImageJ.

### Phagocytosis

Neutrophils (0.3 × 10^6^) were activated with Zy-FITC/op (100 µg/mL) for 20 min at 37 °C and then washed with PBS. Fluorescence of the samples was measured in a flow cytometer (FACSVerse™, BD Biosciences) before and after the addition of the quenching solution (0.4% Trypan blue in PBS citrate, pH 4.4), as described by Nuutila and collaborators^50^, with modifications. Ten thousand cells were analysed.

### ROS production

ROS production was measured by luminol-dependent chemiluminescence (CL) assay as described by Kanashiro and collaborators^51^. Briefly, neutrophils (0.2  × 10^6^) were pre-treated (when indicated) for 1 h with oxalate (3 mM, Sigma, 71800), TEPP-46 (30 µM, Millipore, 5054870001), 2-Deoxy-D-glucose (2-DG, 3 mM, Sigma, D8375), 3PO (10 µM, Calbiochem, 525330), phosphoenolpyruvate (PEP, 1 mM, Sigma, P0564), 5-pentadecylresorcinol (5-PDR, 30 µM, Sigma, 91822) or propranolol (30 µM, Sigma, P0884). Neutrophils were then activated with Zy/op (100 µg/mL) or *S. aureus* (MOI = 3) in the presence of luminol (Sigma, A8511) 10^−4 ^M or lucigenin (Sigma, M8010). The reaction was monitored in a luminometer (FlexStation 3 Multi-Mode Microplate Reader, Molecular Devices, San Jose, CA) for 1 h, at 37 °C, and the results were expressed as the area under the time-course CL curve (AUC).

### Killing assays

Neutrophils (1 × 10^6^) were exposed to *S. aureus* (3 × 10^6^) opsonised with serum (MOI = 3) for 2 h, at 37 °C and 5% CO_2_. For human samples, neutrophils were pre-treated with oxalate (3 mM) or TEPP-46 (30 µM) for 1 h before being exposed to *S. aureus*. Cells were centrifuged and the supernatant discarded. Neutrophils were lysed by adding distilled H_2_O. The lysates were serially diluted in PBS and plated on Mueller-Hinton agar plates and incubated overnight at 37 °C. The results were expressed by CFU per mL.

### Neutrophil extracellular trap assay

This procedure was performed as previously described^45^. Briefly, plasma or culture supernatant was added to a 96-well clear-bottom black plate coated with anti-MPO antibody (5 μg/ml, Thermo Fisher Scientific, PA5-16672) and incubated overnight at 4 °C. The amount of DNA bound to MPO was quantified using the Quant-iT™ PicoGreen® kit (Invitrogen, P11496). The fluorescence intensity (excitation at 488 nm and emission at 525 nm) was quantified by a FlexStation 3 Microplate Reader (Molecular Devices, CA, USA).

### Biochemical parameters

Neutrophils (0.2 × 10^6^) were activated with Zy/op (100 µg/mL) for 6 h, at 37 °C and 5% CO_2_. Lactate concentration in cultured supernatant was measured with the Enzymatic Lactate Kit (Labtest, 138).

For Dihydroxyacetone Phosphate, Glycerol-3-Phosphate, Triose Phosphate Isomerase, NADP/NDPH, glutathione (GSH/GSSG/Total), Pyruvate Kinase activity and Pyruvate analysis, we used commercial kits. Briefly, murine or human neutrophils (1 × 10^6^) were activated with Zy/op (100 µg/mL) for 1 h, at 37 °C and 5% CO_2_. For human samples, neutrophils were pre-treated for 1 h with oxalate (3 mM) before being activated with Zy/op. Samples were centrifuged and prepared according to manufacturer’s instructions. The following kits were used: PicoProbe™ Dihydroxyacetone Phosphate (DHAP) Fluorometric Assay Kit, Glycerol-3-Phosphate (G3P) Colorimetric Assay Kit, Triose Phosphate Isomerase (TPI) Activity Colorimetric Assay Kit, NAD/NADH Quantitation Colorimetric Kit, Glutathione (GSH/GSSG/Total) Fluorometric Assay Kit and Pyruvate Kinase Activity Colorimetric/Fluorometric Assay kit (Biovision, K673, K641, K670, K337 and K709, respectively); and Pyruvate Assay kit (Cayman, 700470).

### Glucose tracing analysis

Neutrophils were activated with Zy/op at 37 °C for 1 h in the presence of D-(+)-Glucose-^13^C_6_ (Cayman, 26707) under slow rotation. Samples were centrifuged and pellets were extracted with 200 µL of methanol/water (8:2 v:v), and centrifuged at 21,000 × *g*, 4 °C, for 10 min (Boeco M-240R, Germany). The supernatant (100 µL) was evaporated up to dryness (Analitica Christ RVC2-18, Sao Paulo, Brazil) and the residue was reconstituted in 80 µL of water. The analysis was performed using an Acquity UPLC-TQD MS/MS System (Waters Corp., Massachusetts, EUA) and metabolites were separated on a Kinetex F5 column 50 mm × 2.1 mm, 1.7 µm (Phenomenex, California, EUA) with a pre-column of the same material, maintained at 40 °C. The mobile phase was composed by solvent A (10 mM tributylamine and 15 mM acetic acid in water) and B (methanol and water, 8:2 v:v). The elution program was as follows: 1% B (initial), 1-10% B (3 min), 10–90% B (4 min), 1% B (4.1 min), 1% B (6.5 min). The flow rate was 0.4 mL.min^−1^, the injection volume was 5 µL in full loop mode, and the autosampler temperature was set to 15 °C. The mass spectrometry (MS) was operated in the negative ion and selected ion recording (SIR) mode using an electrospray voltage of 2.5 kV, source temperature of 150 °C and desolvation temperature of 500 °C. Nitrogen was used as desolvation gas (1000 L h^−1^) and as a cone gas (50 L h^−1^). Identification of isotopically labelled metabolites was based on its unit resolution *m/z* and retention time corresponding to unlabelled metabolites determined with chemical standards (Sigma). The analyses were performed using MassLynx V 4.1 Software (Waters Corp.) and the abundance of labelled glycolytic intermediates was determined by the peak height.

### Flow cytometry analysis

Neutrophils (0.3 × 10^6^) were activated with Zy/op (100 µg/mL) for 1 h, at 37 °C and 5% CO_2_. Neutrophils were washed in PBS and incubated with MitoSOX (5 µM, Thermo, M36008) or Mitotracker Red (50 nM, Thermo, M7512) and Green (100 nM, Thermo, M7514) for 30 min. Samples were washed twice in PBS and immediately analysed by flow cytometry.

For glucose uptake assay, neutrophils (0.5 × 10^6^) in glucose-free RPMI medium supplemented with the fluorescent glucose analogue 2-deoxy-2-[(7-nitro-2,1,3-benzoxadiazol-4-yl)amino]-D-glucose (2-NBDG, Invitrogen, 30 µM, N13195) were activated with Zy/op (100 µg/mL) for 30 min, at 37 °C and 5% CO_2_. Neutrophils were washed twice in PBS and immediately analysed by flow cytometry.

To evaluate the expression of surface antigens, neutrophils were incubated with specific antibodies to GLUT1 (1:200, Abcam, Cat# ab209449), Ly6G (1:200, BD Bioscience, Cat# 560599) CD15 (1:100, BD, Cat# 562370) or CD11b (1:200, Biolegend, Cat# 101212) or the appropriate isotype controls for 1 h. Viable cells were assessed by incubating cells with Fixable Viability Dye (Thermo).

The fluorescence of samples was measured in a flow cytometer (FACSVerse™, BD Biosciences) and analysed using FlowJo software (Tree Star). Ten thousand cells were analysed^52^.

### Analysis of ECAR and OCR with the Seahorse XF Platform

To evaluate the extracellular acidification rate (ECAR) and the oxygen consumption rate (OCR), neutrophils (0.1 × 10^6^) in Seahorse XF RPMI Medium without glucose (Agilent) were seeded in a 96-well XF Cell Culture Microplate (Agilent) pre-coated with poly-L-lysine. Cells were left to adhere for one hour at 37 °C without CO_2_. Neutrophils were activated with Zy/op (100 µg/mL), and ECAR and OCR were then measured on a Seahorse XF Analyser (Agilent) in basal conditions, and upon the sequential addition of glucose (5.56 mM), Rotenone/Antimycin (5 µM), and 2-deoxyglucose (2-DG; 50 mM).

#### Quantification and statistical analysis

Prism 9.2.0 software (GraphPad) was used for data analysis. Comparisons for two groups were carried out using unpaired two-tailed Student’s *t* tests, and one-way ANOVA with Tukey’s post hoc test for multiple comparisons. Comparisons for the time course of skin infection were performed using two-way ANOVA with Bonferroni’s post hoc test. Data are represented as mean ± SEM or bacterial load as a median. Statistical significance: *p < 0.05.

### Reporting summary

Further information on research design is available in the Nature Portfolio Reporting Summary linked to this article.

## Supplementary information

Supplementary information

Peer Review File

Reporting Summary

## Data Availability

All data generated or analysed during this study are included in this published article (and its supplementary information files). Data sharing not applicable to this article as no datasets were generated or analysed during the current study. Source data are provided with this paper.

## Source data

Source data
