# Supplementary material for: The metabolic function of pyruvate kinase M2 regulates reactive oxygen species production and microbial killing by neutrophils
Source: Nat Commun. 2023 Jul 17;14:4280. doi: 10.1038/s41467-023-40021-6 (PMC10352279; doi:10.1038/s41467-023-40021-6)
Supplement: Supplementary file 1 — Supplementary information [file 41467_2023_40021_MOESM1_ESM.pdf]

SUPPLEMENTARY INFORMATION

The metabolic function of pyruvate kinase M2 regulates reactive oxygen species production and microbial killing by neutrophils

Juliana Escher Toller-Kawahisa,[1,2*] Carlos Hiroji Hiroki,[1,2] Camila Meirelles de Souza Silva,[1,2] Daniele Carvalho Nascimento,[1,2] Gabriel Azevedo Públio,[1,2] Timna Varela Martins,[1,2] Luis Eduardo Alves Damasceno,[1,2] Flávio Protásio Veras,[1,2] Paula Ramos Viacava,[1,2] Fábio Yuji Sukesada,[1] Emily Anne Day,[3] Alessia Zotta,[3] Tristram Alexander Jasper Ryan,[3] Rodrigo Moreira da Silva,[4] Thiago Mattar Cunha,[1,2] Norberto Peporine Lopes,[4] Fernando de Queiroz Cunha,[1,2] Luke Anthony John O'Neill,[3] José Carlos Alves-Filho.[1,2*]

[1]Department of Pharmacology, Ribeirao Preto Medical School, University of Sao Paulo, Ribeirao Preto, Brazil.

[2]Center for Research in Inflammatory Diseases, Ribeirao Preto Medical School, University of Sao Paulo, Ribeirao Preto, Brazil.

[3]School of Biochemistry and Immunology, Trinity Biomedical Science Institute, Trinity College Dublin, Dublin 2, Ireland.

[4]NPPNS, Department of Biomolecular Sciences, School of Pharmaceutical Sciences of Ribeirao Preto, University of São Paulo, Ribeirao Preto, Brazil.

*Correspondence: jutoller@hotmail.com (J.E.T.-K.), jcafilho@usp.br (J.C.A.-F.)

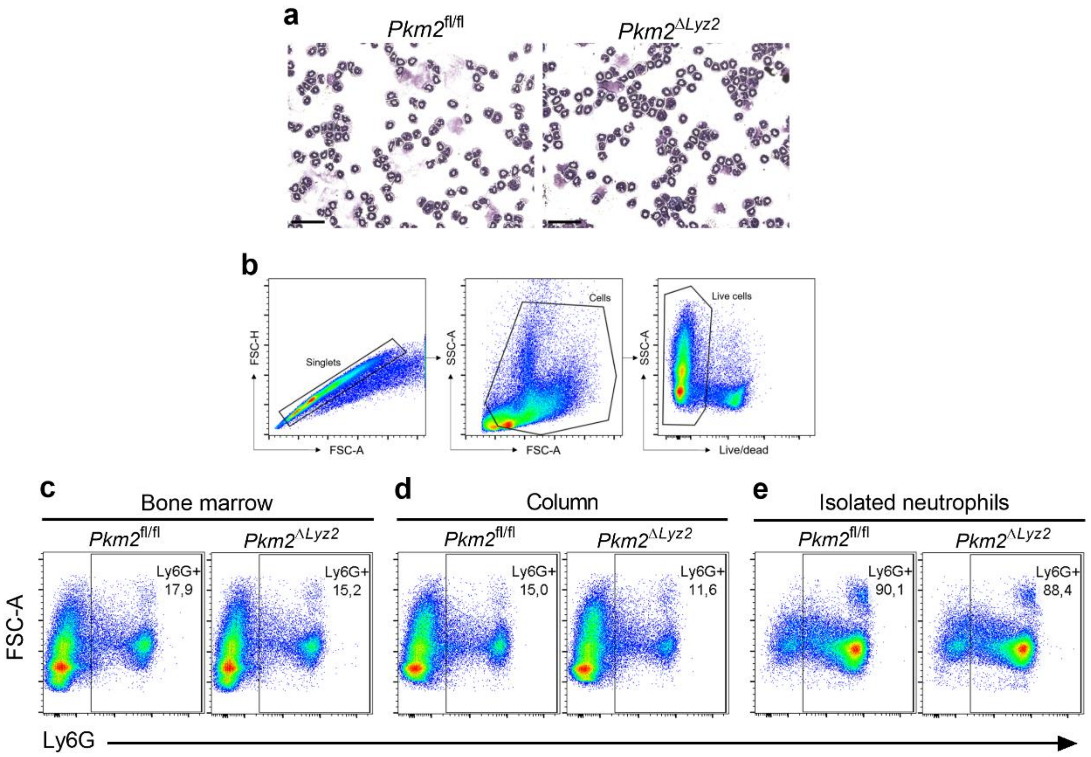

Supplementary figure 1 │ Efficacy of neutrophil isolation method. a, Representative pictures showing the efficacy of neutrophils isolation from bone marrow of wild-type (*Pkm2*$^{fl/fl}$) and PKM2-deficient (*Pkm2*$^{\Delta Lyz2}$) mice. Morphological features were evaluated by a light microscope at 40X magnification. The scale bar indicates 12 µm. **b**, Representative gating strategies for neutrophils (Ly6G^{+}). Doublets were excluded by FSC-H and FSC-A gating for all flow cytometry analyses. We negatively selected live cells. **c**, Representative flow cytometry plots showing the frequency of neutrophils in total bone marrow of *Pkm2*$^{fl/fl}$ and *Pkm2*$^{\Delta Lyz2}$ mice. **d**, Representative flow cytometry plots showing the frequency of neutrophils that are retained in the column and are lost during the isolation process. **e**, Frequency of neutrophils recovered after the isolation process. Representative of 2 independent experiments.

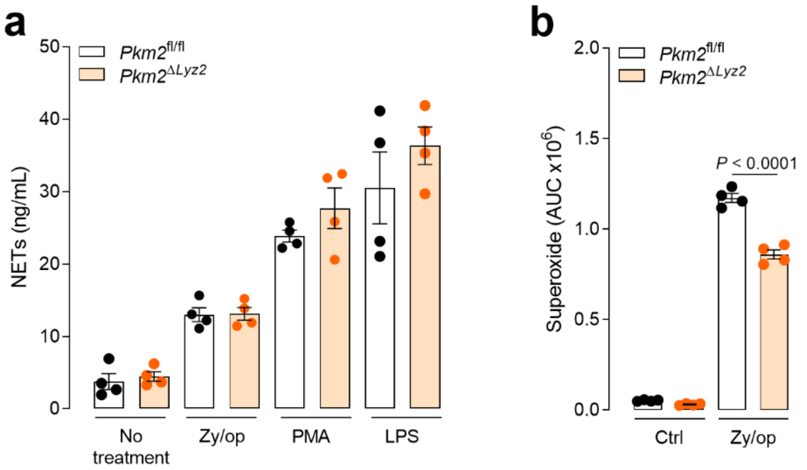

Supplementary figure 2 │ Effect of PKM2 on neutrophils functions. a, NET production by wild-type (*Pkm2*$^{fl/fl}$) and PKM2-deficient (*Pkm2*$^{\Delta Lyz2}$) neutrophils activated with Zy/op (100 µg/mL), Phorbol myristate acetate (50 nM, PMA) or LPS (10 µg/mL) for 4h. n=4 biologically independent wild-type and PKM2-deficient neutrophils. Representative of 1 independent experiment **b**, Superoxide production by wild-type and PKM2-deficient neutrophils activated with Zy/op for 1h. n=4 biologically independent wild-type and PKM2-deficient neutrophils. Representative of 2 independent experiments. Error bars are mean ± SEM. p values were determined by one-way ANOVA followed by Tukey's post hoc test. Source data are provided in the Source data file.

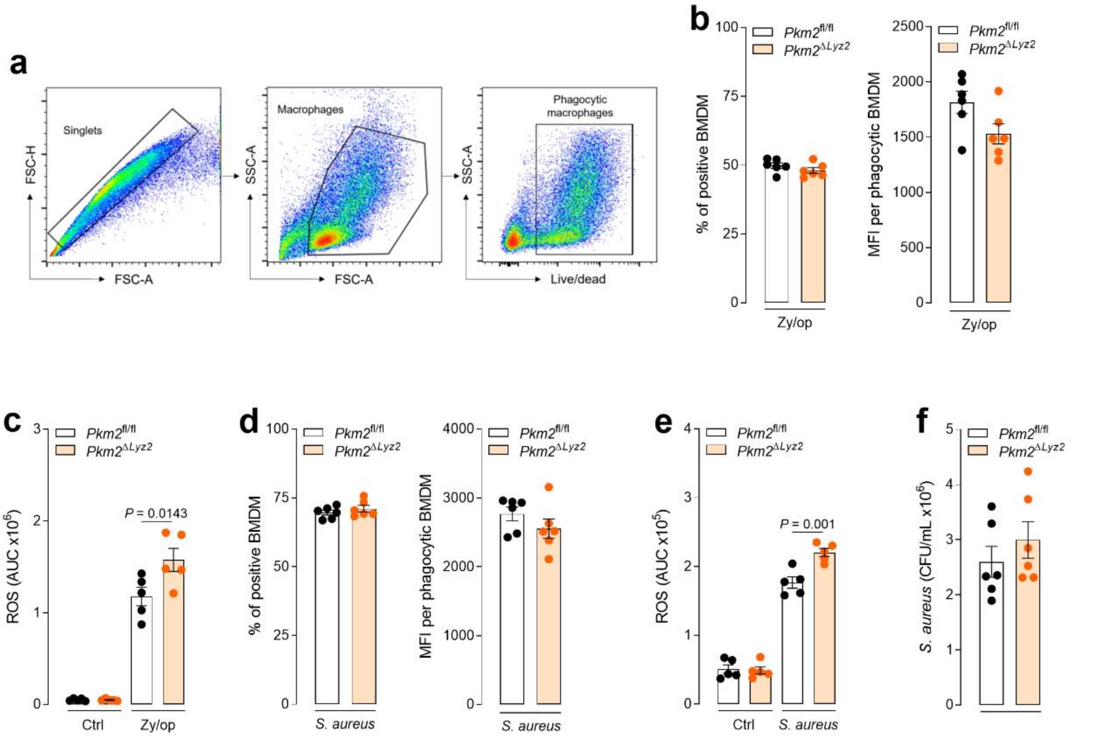

Supplementary figure 3 │ Effect of PKM2 on bone marrow-derived macrophages (BMDM) functions. a, Representative gating strategies for macrophages. Doublets were excluded by FSC-H and FSC-A gating for all flow cytometry analyses. We negatively selected live cells. **b**, Phagocytosis of Zy/op-FITC (100 µg/mL) by wild-type (Pkm2$^{fl/fl}$) and PKM2-deficient (Pkm2$^{\Delta Lyz2}$) BMDMs determined by flow cytometry. n=6 biologically independent wild-type and PKM2-deficient BMDMs. Representative of 2 independent experiments. **c**, ROS production by murine BMDMs activated with Zy/op determined by luminol-dependent chemiluminescence assay. Representative area under the curve (AUC) graph bar. n=5 biologically independent wild-type and PKM2-deficient BMDMs. Representative of 2 independent experiments. **d**, Phagocytosis of *S. aureus* (MOI=3) by murine BMDMs determined by flow cytometry. n=6 biologically independent wild-type and PKM2-deficient BMDMs. Representative of 2 independent experiments. **e**, ROS production by murine BMDMs activated with *S. aureus* determined by luminol-dependent chemiluminescence assay. Representative area under the curve (AUC) graph bar. n=5 biologically independent wild-type and PKM2-deficient BMDMs. Representative of 2 independent experiments. **f**, Number of viable *S. aureus* recovered after 2h from lysates of BMDMs. n=6 biologically independent wild-type and PKM2-deficient BMDMs. Representative of 2 independent experiments. Error bars are mean ± SEM. p values were determined by two-tailed unpaired Student's t-test (a, c, e) or one-way ANOVA followed by Tukey's post hoc test (b, d). Source data are provided in the Source data file.

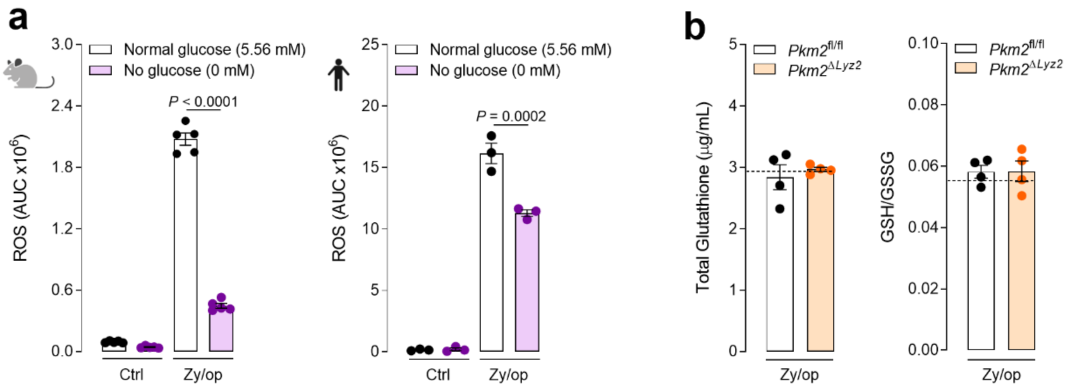

Supplementary figure 4 │ Glucose supports ROS production. a, ROS production by wild-type mouse and human neutrophils activated with Zy/op (100 µg/mL) for 1h in the presence or absence of glucose. n=5 biologically independent murine neutrophils (representative of 2 independent experiments) and n=3 technical replicates (representative of 2 independent experiments), respectively. **b**, Total glutathione and GSH/GSSG ratio concentrations in wild-type and PKM2-deficient neutrophils activated with Zy/op for 1h. n=4 biologically independent murine neutrophils. Representative of 2 independent experiments. Error bars are mean ± SEM. p values were determined one-way ANOVA followed by Tukey's post hoc test (a) or two-tailed unpaired Student's t-test (b). Clip art provided by Biorender. Source data are provided in the Source data file.

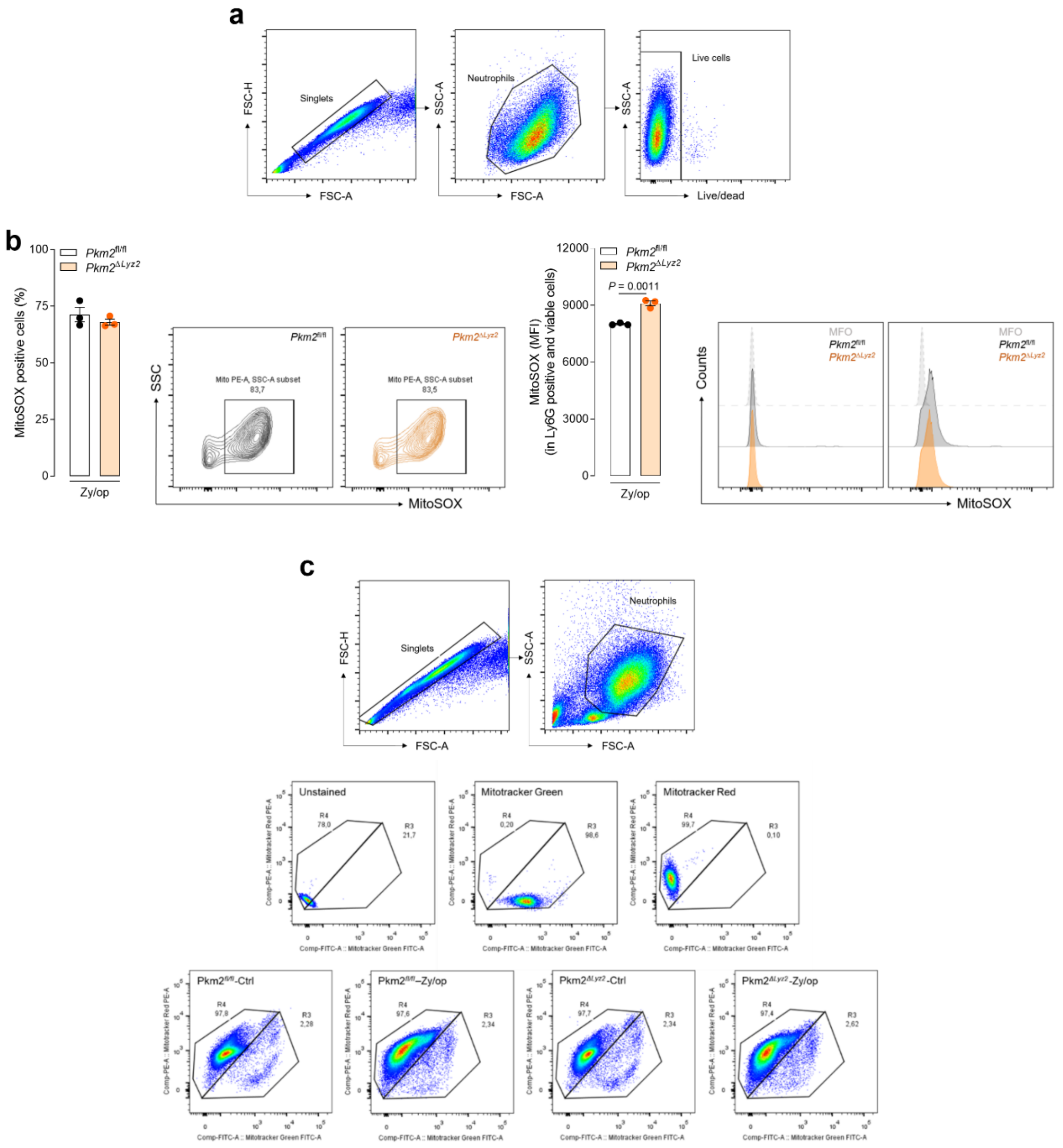

Supplementary figure 5 │ PKM2 has no effect on mitochondrial function. a, Representative gating strategies for neutrophils. Doublets were excluded by FSC-H and FSC-A gating for all flow cytometry analyses. Viable cells were negatively selected. **b,** Representative flow cytometry dot plots, histogram and respective graphs bar showing the frequency and the median fluorescence intensity (MFI) of mitochondrial ROS production in wild-type (*Pkm2*$^{fl/fl}$) and PKM2-deficient (*Pkm2*$^{\Delta Lyz2}$) neutrophils activated in vitro with Zy/op-FITC (100 µg/mL) for 1h determined by flow cytometry. n=3 biologically independent wild-type and PKM2-deficient murine neutrophils. Representative of 2 independent experiments. **c**, Representative dot plots showing the mitochondrial function evaluated by Mitotracker Red and Green. Representative of 1 independent experiment. Error bars are mean ± SEM. p values were determined by two-tailed unpaired Student’s t-test (b). Source data are provided in the Source data file.

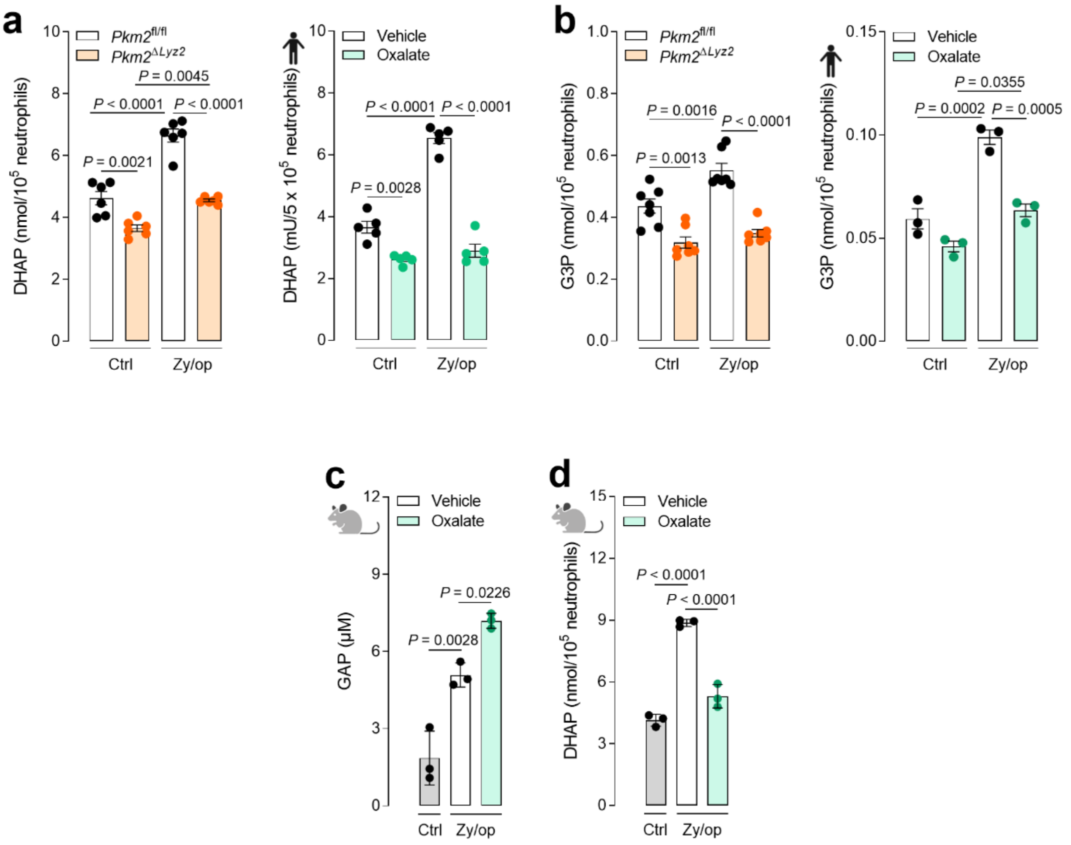

Supplementary figure 6 │ PKM2 modulates the DHAP pathway. a, DHAP production by wild-type ($Pkm2^{fl/fl}$) and PKM2-deficient ($Pkm2^{\Delta Lyz2}$) neutrophils and in human neutrophils pre-treated with oxalate for 1h and activated with Zy/op (100 µg/mL) for 1h. n=6 biologically independent murine neutrophils (representative of 2 independent experiments) and n=5 independent donors (representative of 5 pooled independent experiments), respectively. Representative of 2 independent experiments **b,** G3P production by wild-type and PKM2-deficient neutrophils and in human neutrophils pre-treated with oxalate for 1h and activated with Zy/op for 1h. n = 7 biologically independent murine neutrophils (representative of 2 independent experiments) and n=3 independent donors (representative of 3 pooled independent experiments), respectively. **c,** GAP production by wild-type neutrophils pre-treated with oxalate for 1h and activated with Zy/op for 1h. n=3 biologically independent murine neutrophils. Representative of 2 independent experiments. **d,** DHAP production by wild-type neutrophils pre-treated with oxalate for 1h and activated with Zy/op for 1h. n = 3 biologically independent murine neutrophils. Representative of 2 independent experiments. Error bars are mean ± SEM. p values were determined by one-way ANOVA followed by Tukey's post hoc test. Clip art provided by Biorender. Source data are provided in the Source data file.

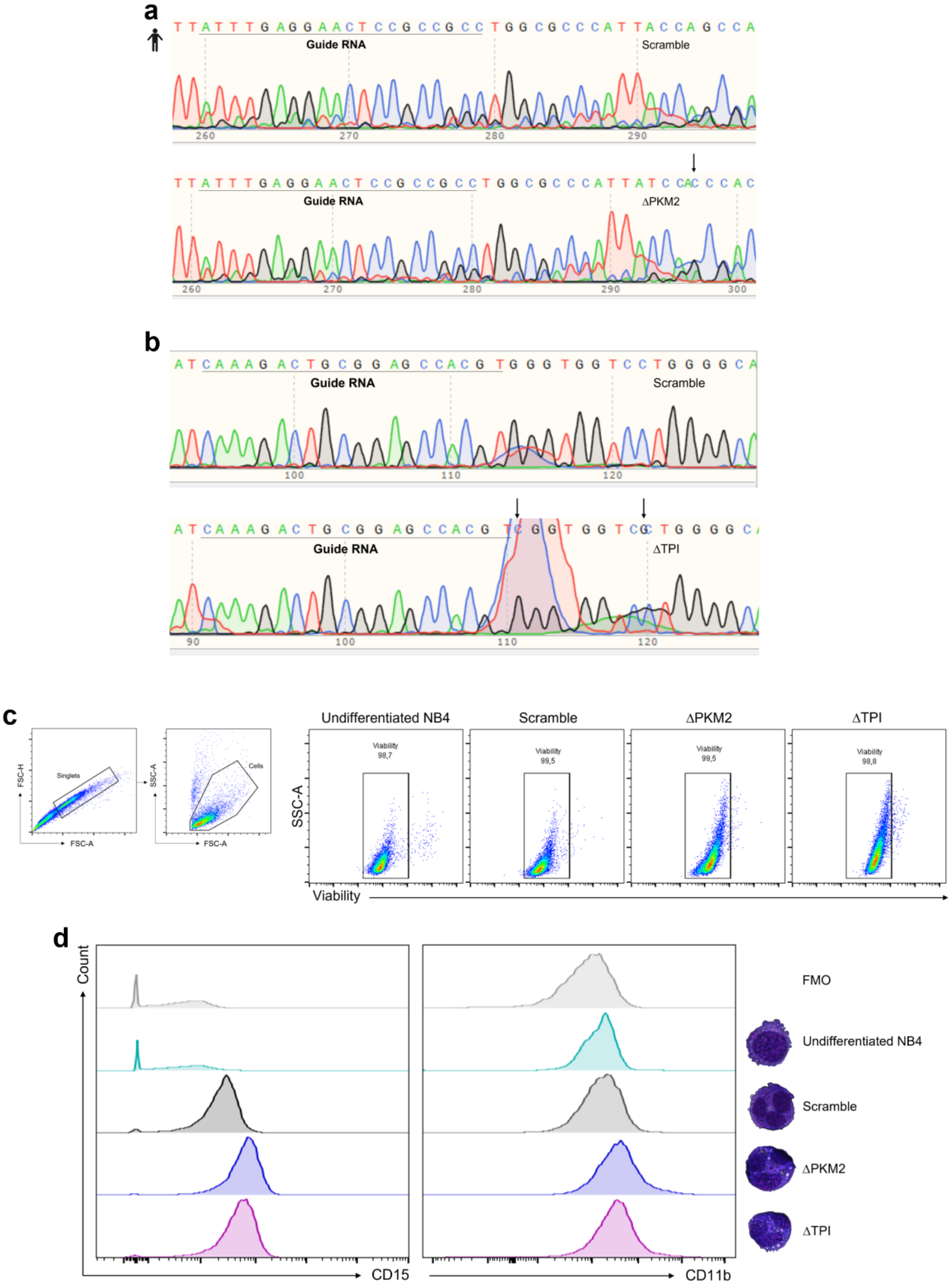

Supplementary figure 7 │ CRISPR/Cas9-based disrupts *Tpi* and *Pkm2*. Sequencing of **a**, *Pkm2* gene in NB4/PKM2-KO cells and **b**, *Tpi* gene in NB4/TPI-KO cells targeted by sgPKM2 and sgTPI, respectively. **c**, Representative flow cytometry plots showing the viability of NB4 cells differentiated with ATRA and that had *Pkm2* and *Tpi* disruption by CRISPR/Cas9-based editing. **d**, Representative histogram of CD15 and CD11b expression in NB4 cells determined by flow cytometry. Representative of 2 independent experiments. Clip art provided by Biorender. Source data are provided in the Source data file.
