# Supplementary material for: The metabolic function of pyruvate kinase M2 regulates reactive oxygen species production and microbial killing by neutrophils
Source: Nat Commun. 2023 Jul 17;14:4280. doi: 10.1038/s41467-023-40021-6 (PMC10352279; doi:10.1038/s41467-023-40021-6)
Supplement: Supplementary file 3 — Reporting Summary [file 41467_2023_40021_MOESM3_ESM.pdf]

nature portfolio

Corresponding author(s): José Carlos Alves Filho

Last updated by author(s): Jun 12, 2023

Reporting Summary

Nature Portfolio wishes to improve the reproducibility of the work that we publish. This form provides structure for consistency and transparency in reporting. For further information on Nature Portfolio policies, see our Editorial Policies and the Editorial Policy Checklist.

Statistics

For all statistical analyses, confirm that the following items are present in the figure legend, table legend, main text, or Methods section.

n/a	Confirmed	
☐	☒	The exact sample size (*n*) for each experimental group/condition, given as a discrete number and unit of measurement
☐	☒	A statement on whether measurements were taken from distinct samples or whether the same sample was measured repeatedly
☐	☒	The statistical test(s) used AND whether they are one- or two-sided<br>*Only common tests should be described solely by name; describe more complex techniques in the Methods section.*
☒	☐	A description of all covariates tested
☐	☒	A description of any assumptions or corrections, such as tests of normality and adjustment for multiple comparisons
☐	☒	A full description of the statistical parameters including central tendency (e.g. means) or other basic estimates (e.g. regression coefficient) AND variation (e.g. standard deviation) or associated estimates of uncertainty (e.g. confidence intervals)
☐	☒	For null hypothesis testing, the test statistic (e.g. *F*, *t*, *r*) with confidence intervals, effect sizes, degrees of freedom and *P* value noted<br>*Give P values as exact values whenever suitable.*
☒	☐	For Bayesian analysis, information on the choice of priors and Markov chain Monte Carlo settings
☒	☐	For hierarchical and complex designs, identification of the appropriate level for tests and full reporting of outcomes
☒	☐	Estimates of effect sizes (e.g. Cohen's *d*, Pearson's *r*), indicating how they were calculated

Our web collection on statistics for biologists contains articles on many of the points above.

Software and code

Policy information about availability of computer code

Data collection	Leica LAS X Life Science Microscope Software, ChemiDoc XRS Imager, Agilent Seahorse Wave (2.4.3), FCS Express V3 (De Novo Software), MassLynx V 4.1 Software.
Data analysis	GraphPad Prism 9.2.0, FlowJo v10, Image Lab 6.0.1, ImageJ (1.53t)

For manuscripts utilizing custom algorithms or software that are central to the research but not yet described in published literature, software must be made available to editors and reviewers. We strongly encourage code deposition in a community repository (e.g. GitHub). See the Nature Portfolio guidelines for submitting code & software for further information.

Data

Policy information about availability of data

All manuscripts must include a data availability statement. This statement should provide the following information, where applicable:

- Accession codes, unique identifiers, or web links for publicly available datasets
- A description of any restrictions on data availability
- For clinical datasets or third party data, please ensure that the statement adheres to our policy

All data generated or analysed during this study are included in this published article (and its supplementary information files). Source data are provided with this paper. Data sharing not applicable to this article as no datasets were generated or analysed during the current study.

Research involving human participants, their data, or biological material

Policy information about studies with human participants or human data. See also policy information about sex, gender (identity/presentation), and sexual orientation and race, ethnicity and racism.

Reporting on sex and gender	Thirty healthy donors of both sexes with ages between 18-40 years were included in this study.
Reporting on race, ethnicity, or other socially relevant groupings	All the participants were Brazilians and inhabited the same geographic region.
Population characteristics	The exclusion criteria were smoking, obesity, diabetes, pregnancy and anti-inflammatory use.
Recruitment	Individuals were recruited by email and belong to the comunity of the Sao Paulo University, Ribeirao Preto campus. Individuals were selected accordingly to the exclusion criteria.
Ethics oversight	All recruited volunteers provided written informed consent form. The study was approved by the Human Subjects Institutional Committee of the Ribeirão Preto Medical School, Brazil (License number: 31033519.4.0000.5440).

Note that full information on the approval of the study protocol must also be provided in the manuscript.

Field-specific reporting

Please select the one below that is the best fit for your research. If you are not sure, read the appropriate sections before making your selection.

[x] Life sciences [ ] Behavioural & social sciences [ ] Ecological, evolutionary & environmental sciences

For a reference copy of the document with all sections, see nature.com/documents/nr-reporting-summary-flat.pdf

Life sciences study design

All studies must disclose on these points even when the disclosure is negative.

Sample size	Sample size was determined based on previous experience or similar published studies. We have used at least 1 biological replicate for each individual experiment, which was performed on 3 independent occasions unless otherwise stated. Although we did not use statistical methods to calculate sample size, we decided to use a minimun of 3 biological replicates per experiment to account for biological variability, taking into account the fact that the majority of experiments were performed in primiry murine neutrophils from inbred mice.
Data exclusions	No data were excluded.
Replication	Experiments in vitro were reproducible and were repeated in 3 different occasions, unless stated otherwise. The in vivo experiments were performed on two different occasions and were also reproducible.
Randomization	The allocation of samples and mice into treatments groups was random. For the glucose tracing, the samples were processed in random order and experimenters were blinded to experimental conditions.
Blinding	Investigators were blinded for image analysis, glucose tracing analysis and group allocation for in vivo experiments. For In vitro and in vivo experiments were not blinded due the lack of available experimenters with required expertise.

Reporting for specific materials, systems and methods

We require information from authors about some types of materials, experimental systems and methods used in many studies. Here, indicate whether each material, system or method listed is relevant to your study. If you are not sure if a list item applies to your research, read the appropriate section before selecting a response.

Materials & experimental systems

n/a	Involved in the study	
[ ]	[x]	Antibodies
[ ]	[x]	Eukaryotic cell lines
[x]	[ ]	Palaeontology and archaeology
[ ]	[x]	Animals and other organisms
[ ]	[x]	Clinical data
[x]	[ ]	Dual use research of concern
[x]	[ ]	Plants

Methods

n/a	Involved in the study	
[x]	[ ]	ChIP-seq
[ ]	[x]	Flow cytometry
[x]	[ ]	MRI-based neuroimaging

Antibodies

Antibodies used

For Western Blotting:
Anti-PKM2 (Clone D78A4) Cell Signalling Cat# 4053P
Mouse Anti-gp91[phox] (Clone 53/gp91[phox]) BD Transduction Cat# 611414
Anti-p47-phox (polyclonal) Millipore Cat# 07-500
Anti-Phospho-p47-phox (Ser370) (polyclonal) Invitrogen Cat# PA5-36863
Anti-B-actin (clone 8H10D10) Cell signalling Cat# 3700S
Anti-rabbit IgG HRP Sigma-Aldrich Cat# A0545
Anti-mouse IgG HRP Sigma-Aldrich Cat# A9044

For Immunostaining:
Anti-rabbit IgG AlexaFluor 488 Abcam Cat# ab150065
Anti-GLUT1-PE (clone EPR3915) Abcam Cat# ab209449
Anti-Ly6G-APC (clone 1A8) BD Biosciences Cat# 560599
Anti-mouse/human CD11b (clone M1/70) Biolegend Cat# 101202
Anti-human CD15 (clone W6D3) BD Biosciences Cat# 562370
Anti-IgG H&L-Alexa Fluor 488 (polyclonal) Abcam Cat# ab150157

For neutrophil isolation:
Neutrophil Isolation kit Microbeads Miltenyi Biotech Cat# 130-097-658

Validation

All antibodies were from a commercial vendors and validated by the companies. Antibodies were used according to validation listed in manufacturer's instructions.
For Western Blotting:
Anti-PKM2 (Clone D78A4) Cell Signalling Cat# 4053P / Reactivity: H M Rb Mk / Application validation WB, IP, IHC-P, IF-IC, FC-FP
Mouse Anti-gp91[phox] (Clone 53/gp91[phox]) BD Transduction Cat# 611414 / Reactivity: M Rb / Application validation WB, IF-IC
Anti-p47-phox (polyclonal) Millipore Cat# 07-500 / Reactivity: M / Application validation WB
Anti-Phospho-p47-phox (Ser370) (polyclonal) Invitrogen Cat# PA5-36863 / Reactivity: H M Rb / Application validation WB, IP, IHC-P, IF-IC
Anti-B-actin (clone 8H10D10) Cell signalling Cat# 3700S / Reactivity: H M Rb Mk Hm Dg / Application validation WB, IHC-P, IF-IC, FC-FP
Anti-rabbit IgG HRP Sigma-Aldrich Cat# A0545 / Reactivity: Rb / Application validation WB
Anti-mouse IgG HRP Sigma-Aldrich Cat# A9044 / Reactivity: M / Application validation WB

For Immunostaining:
Anti-rabbit IgG AlexaFluor 488 Abcam Cat# ab150065 / Reactivity: Rb / Application validation ICC/IF, ELISA, Flow Cyt, IHC-P, IHC-Fr
Anti-GLUT1-PE (clone EPR3915) Abcam Cat# ab209449 / Reactivity: H M R / Application validation ICC/IF, Flow Cyt
Anti-Ly6G-APC (clone 1A8) BD Biosciences Cat# 560599 / Reactivity: M / Application validation Flow Cyt
Anti-mouse/human CD11b (clone M1/70) Biolegend Cat# 101202 / Reactivity: M H Cy Mk / Application validation Flow Cyt, IHC-F, IP
Anti-human CD15 (clone W6D3) BD Biosciences Cat# 562370 / Reactivity: H / Application validation Flow Cyt
Anti-IgG H&L-Alexa Fluor 488 (polyclonal) Abcam Cat# ab150157 / Reactivity: R / Application validation Flow Cyt, IHC-Fr, IHC-P, ICC/IF, ELISA

Eukaryotic cell lines

Policy information about cell lines and Sex and Gender in Research

Cell line source(s)	NB4 cells (ATCC)
Authentication	Authentication of NB4 was not performed
Mycoplasma contamination	NB4 cells were mycoplasma-free
Commonly misidentified lines (See ICLAC register)	No commonly misidentified lines were used

Animals and other research organisms

Policy information about studies involving animals; ARRIVE guidelines recommended for reporting animal research, and Sex and Gender in Research

Laboratory animals

C57BL/6 wild-type mice were purchased from Charles River. Lyz2Cre (strain# 004781) and PKM2flox/flox (strain# 024048) mice were obtained from Jackson Laboratories. Myeloid cell-(Pkm2ΔLyz2) specific-Pkm2-deficient mice were generated by crossing the Pkm2flox/flox mice with Lyz2Cre mice on a C57BL/6 background. Pkm2fl/fl mice without Lyz2Cre gene were used as controls for all experiments. All experiments were carried out with 6–7-week-old female and male littermate mice. The protocols used for animal experimentation were approved by the Animal Welfare Committee of the Ribeirão Preto Medical School, University of São Paulo (protocol number: 143/2017). Animals were bred and maintained under specific pathogen-free conditions (12/12-hour light/dark cycle, 55 ± 5% humidity, 21-23°C) at the animal facility of the Ribeirão Preto Medical School, University of São Paulo. Mice were randomly assigned to experimental groups. Mice were euthanized with Xylazine/Ketamine (10 mg/kg / 80 mg/kg) administrated

intraperitoneally (i.p.).

Wild animals	No wild animals were used.
Reporting on sex	All experiments were carried out with 6–7-week-old female and male littermate mice. There were no differences between female and male mice in vitro or in vivo.
Field-collected samples	No field-collection samples were used.
Ethics oversight	The protocols used for animal experimentation were approved by the Animal Welfare Committee of the Ribeirão Preto Medical School, University of São Paulo (protocol number: 143/2017).

Note that full information on the approval of the study protocol must also be provided in the manuscript.

Clinical data

Policy information about clinical studies

All manuscripts should comply with the ICMJE guidelines for publication of clinical research and a completed CONSORT checklist must be included with all submissions.

Clinical trial registration	*Provide the trial registration number from ClinicalTrials.gov or an equivalent agency.*
Study protocol	*Note where the full trial protocol can be accessed OR if not available, explain why.*
Data collection	*Describe the settings and locales of data collection, noting the time periods of recruitment and data collection.*
Outcomes	*Describe how you pre-defined primary and secondary outcome measures and how you assessed these measures.*

Flow Cytometry

Plots

Confirm that:

- [x] The axis labels state the marker and fluorochrome used (e.g. CD4-FITC).
- [x] The axis scales are clearly visible. Include numbers along axes only for bottom left plot of group (a 'group' is an analysis of identical markers).
- [x] All plots are contour plots with outliers or pseudocolor plots.
- [x] A numerical value for number of cells or percentage (with statistics) is provided.

Methodology

Sample preparation	Phagocytosis:<br>Neutrophils (0.3 x 106) were activated with Zy-FITC/op (100 µg/mL) for 20 min at 37 ºC and then washed with PBS. Fluorescence of the samples was measured in a flow cytometer (FACSVerse™, BD Biosciences) before and after the addition of the quenching solution (0.4 % Trypan blue in PBS citrate, pH 4.4), as described by Nuutila and collaborators50, with modifications. Ten thousand cells were analysed.<br>MitoSOX and Mitotracker:<br>Neutrophils (0.3 x 106) were activated with Zy/op (100 µg/mL) for 1h, at 37 °C and 5 % CO2. Neutrophils were washed in PBS and incubated with MitoSOX (5 µM, Thermo, M36008) or Mitotracker Red (50 nM, Thermo, M7512) and Green (100 nM, Thermo, M7514) for 30 min. Samples were washed twice in PBS and immediately analysed by flow cytometry.<br><br>Glucose uptake assay:<br>Neutrophils (0.5 x 106) in glucose-free RPMI medium supplemented with the fluorescent glucose analogue 2-deoxy-2-[(7-nitro-2,1,3-benzoxadiazol-4-yl)amino]-D-glucose (2-NBDG, Invitrogen, 30 µM, N13195) were activated with Zy/op (100 µg/mL) for 30 min, at 37 °C and 5 % CO2. Neutrophils were washed twice in PBS and immediately analysed by flow cytometry.<br><br>Expression of surface antigens:<br>Neutrophils were incubated with specific antibodies to GLUT1 (1:200, Abcam, Cat# ab209449), Ly6G (1:200, BD Bioscience, Cat# 560599) CD15 (1:100, BD, Cat# 562370) or CD11b (1:200, Biolegend, Cat# 101212) or the appropriate isotype controls for 1h. Viable cells were assessed by incubating cells with Fixable Viability Dye (Thermo).
Instrument	FACSVerse™, BD Biosciences
Software	Samples were analysed using FlowJo software (Tree Star).
Cell population abundance	Ten thousand cells were analysed and the purity was around 90%.

Gating strategy	Doublets were excluded by FSC-H and FSC-A gating for all flow cytometry analyses. We negatively selected live cells. Gating strategy is shown in Supplementary Information.

☒ Tick this box to confirm that a figure exemplifying the gating strategy is provided in the Supplementary Information.
